# Classification of painful or painless diabetic peripheral neuropathy and identification of the most powerful predictors using machine learning models in large cross-sectional cohorts

**DOI:** 10.1186/s12911-022-01890-x

**Published:** 2022-05-29

**Authors:** Georgios Baskozos, Andreas C. Themistocleous, Harry L. Hebert, Mathilde M. V. Pascal, Jishi John, Brian C. Callaghan, Helen Laycock, Yelena Granovsky, Geert Crombez, David Yarnitsky, Andrew S. C. Rice, Blair H. Smith, David L. H. Bennett

**Affiliations:** 1 Neural Injury Group, Nuffield Department of Clinical Neuroscience, John Radcliffe Hospital, University of Oxford, Level 6, West Wing, Oxford, OX3 9DU UK; 2 Chronic Pain Research Group, Division of Population Health and Genomics, Mackenzie Building, Ninewells Hospital and Medical School, University of Dundee, Dundee, UK; 3 Department of Neurology, University of Michigan Medical School, Ann Arbor, MI USA; 4 Pain Research, Department of Surgery and Cancer, Faculty of Medicine, Imperial College London, London, UK; 5 Department of Experimental-Clinical and Health Psychology, Faculty of Psychology and Educational Sciences, Ghent University, Ghent, Belgium; 6 Department of Neurology, Rambam Health Care Campus, Technion-Israel Institute of Technology, Haifa, Israel

**Keywords:** Diabetic neuropathy, Neuropathic pain, Machine learning, Risk factors, Predictive modelling

## Abstract

**Background:**

To improve the treatment of painful Diabetic Peripheral Neuropathy (DPN) and associated co-morbidities, a better understanding of the pathophysiology and risk factors for painful DPN is required. Using harmonised cohorts (N = 1230) we have built models that classify painful versus painless DPN using quality of life (EQ5D), lifestyle (smoking, alcohol consumption), demographics (age, gender), personality and psychology traits (anxiety, depression, personality traits), biochemical (HbA1c) and clinical variables (BMI, hospital stay and trauma at young age) as predictors.

**Methods:**

The Random Forest, Adaptive Regression Splines and Naive Bayes machine learning models were trained for classifying painful/painless DPN. Their performance was estimated using cross-validation in large cross-sectional cohorts (N = 935) and externally validated in a large population-based cohort (N = 295). Variables were ranked for importance using model specific metrics and marginal effects of predictors were aggregated and assessed at the global level. Model selection was carried out using the Mathews Correlation Coefficient (MCC) and model performance was quantified in the validation set using MCC, the area under the precision/recall curve (AUPRC) and accuracy.

**Results:**

Random Forest (MCC = 0.28, AUPRC = 0.76) and Adaptive Regression Splines (MCC = 0.29, AUPRC = 0.77) were the best performing models and showed the smallest reduction in performance between the training and validation dataset. EQ5D index, the 10-item personality dimensions, HbA1c, Depression and Anxiety t-scores, age and Body Mass Index were consistently amongst the most powerful predictors in classifying painful vs painless DPN.

**Conclusions:**

Machine learning models trained on large cross-sectional cohorts were able to accurately classify painful or painless DPN on an independent population-based dataset. Painful DPN is associated with more depression, anxiety and certain personality traits. It is also associated with poorer self-reported quality of life, younger age, poor glucose control and high Body Mass Index (BMI). The models showed good performance in realistic conditions in the presence of missing values and noisy datasets. These models can be used either in the clinical context to assist patient stratification based on the risk of painful DPN or return broad risk categories based on user input. Model’s performance and calibration suggest that in both cases they could potentially improve diagnosis and outcomes by changing modifiable factors like BMI and HbA1c control and institute earlier preventive or supportive measures like psychological interventions.

**Supplementary Information:**

The online version contains supplementary material available at 10.1186/s12911-022-01890-x.

## Background

### Peripheral neuropathy as a complication of diabetes

The prevalence of Diabetic Peripheral Neuropathy (DPN) is 29–49% in people with diabetes mellitus [1, 2]. This translates to a global prevalence of ~ 200 million people living with DPN. Up to 50% of patients with DPN will develop chronic neuropathic pain [1, 3]. Painful DPN is characterised by chronic pain that is most severe in the feet [4], but can extend to involve the legs, hands and arms in a typical “glove and stocking distribution”. The pain is often described as a burning sensation, associated with paraesthesiae or dysaesthesiae and occasionally allodynia [4, 5]. The last decade has seen significant advances in our ability to characterise the sensory phenotype of DPN using patient-reported sensory symptoms [6] and standardised quantitative sensory testing [7] which facilitate patient stratification [8].

Painful DPN is strongly associated with poor quality of life and psychological co-morbidities such as depression and anxiety disorders [9, 10]. The mental health burden associated with painful DPN remains an under-recognised and under-treated complication associated with diabetes mellitus. Management of DPN is complicated by several challenges. The condition is under-diagnosed [11], current treatment options are inadequate [3, 4], and we do not understand why some patients with DPN develop pain and others do not.

The pathophysiology of painful DPN is most likely a complex interaction of genetic, environmental and psychological factors. Multiple fundamental neurobiology mechanisms are thought to underly neuropathic pain including hyper-excitability, maladaptive structural plasticity and pro-inflammatory processes within both the peripheral and central nervous system (recently reviewed in [12]). For example, a recent review of studies of the risk factors for neuropathic pain reported that clinical and lifestyle factors such as obesity, poor glucose control, hypertension and neuropathy severity were associated with its presence as well as genetic and psychological factors such as depression and anxiety [13]. Interactions between risk factors may also be important. For example, we have found that the negative impact of glucose control on neuropathy severity is larger in males than in females, whereas stress and anxiety had larger effects in females [8, 14].

In order to improve the treatment of painful DPN and the associated co-morbidities, it is essential that we develop a better understanding of the pathophysiology and risk factors for painful DPN. The DOLORisk project [15] has provided the scientific community with large harmonised datasets that can be exploited in order to better understand the risk factors and build models predicting the development of painful or painless DPN. Moreover, data harmonisation between multiple datasets collected from different centres facilitates estimation of the models performance using cross-validation and rigorous external validation in independent datasets.

### Machine learning

ML is a technique for statistical learning that involves optimisation in order to minimise a loss function and optimise the predictive ability. During training, an ML algorithm learns patterns and determines the optimal values for its internal parameters from data [16, 17]. ML is focused on prediction by optimising the discrimination of different classes; no assumptions about the underlying processes that generate data are required and a well performing model does not replace the rigorous statistical techniques needed to infer causality. The advantage of a well-trained ML model is that it can be easily generalised to predict unobserved outcomes on an unknown dataset. In supervised classification, we train an ML algorithm in a training dataset with known classes (e.g. painful vs painless neuropathy), and use the final model to predict classes in new samples. Reducible prediction errors are bias (i.e. the difference between a model’s prediction and the actual value) and variance (i.e. the difference between predictions of different realizations of the model) [18]. In this context model training involves a trade-off between bias and variance [19, 20]. A model with high bias under-fits the data and a model with high variance over-fits the data. A manifestation of over-fitting is a model that has learned nuances of the training data and cannot be generalised in un-observed data. Thus model validation in an independent test dataset is highly important in order to avoid highly optimistic estimations of real-world model performance.

Another important aspect is how to handle missing data. Every decision taken in training is considered part of the model building and should be benchmarked for its effect on model performance. The same is true for the imputation of missing values in model training, testing and validation. As large clinical cohorts often suffer from missing values in several data points we have developed a framework of utilising multiple imputation of missing values that does not leak information between model training and validation, models the uncertainty introduced due to the imputation, performs outcome agnostic imputation of the validation datasets simulating model deployment and prediction in the presence of missing values [21, 22]. Estimates from multiple imputed datasets are aggregated using Rubin’s rules [22]. Multiple models are then trained in the multiple completed datasets and predictions are aggregated over all the completed instances of the outcome-agnostic imputed validation set. This is a predict-and-aggregate strategy [21, 23]. We have also taken care to encapsulate all model building decisions in a cross-validation approach that ensures no information leakage between in-fold and out-of-fold instances during cross-validation.

Algorithmic modelling and ML techniques are notorious for their “black box” approach that can obscure meaningful relations between predictors and present spurious associations due to chance or systematic errors as related to the outcome. A lot of work has focused on making ML interpretable [24–26]. In the context of a model one can see how changes in predictors influence the model’s predictions [25, 27] and predictors can be ranked based on model specific or model independent metrics. These techniques allow us to explain and better understand the behaviour of ML algorithms. In the rest of the manuscript we will use the words predictors or features interchangeably.

### Predicting diabetes complications

ML has been successfully utilised for predicting [28–30] and developing risk [31–33] equations for diabetes and its complications. Different classifiers have been efficiently utilised providing evidence of good performance for Support Vector Machines (SVM), ensembles of decision trees like Random Forests (RF) and Gradient Boosted Trees, Logistic Regression (LR) models and its extensions and Artificial Neural Networks (ANN). Some models were used to predict/classify diabetes mellitus versus non diabetic people [29, 34–36] and others were developed to predict complications such as cardiovascular diseases, neuropathy and retinopathy, kidney disease or psychological comorbidities [30–32, 37–40]. A recent comprehensive review and meta-analysis [34] considered 23 studies using ML models to predict type 2 diabetes mellitus found that most of them were carried out on cross-sectional cohorts, with sample sizes varied from 234 to 138,146 and with discriminatory indices ranging from 0.72 to 1. However, none of them performed any independent external validation. Ensembles of diverse classifiers including ANN, SVM, Bayesian classifiers and LR outperformed individual algorithms on the prediction and detection of diabetes and are the only ones associated with really high accuracies [41]. Regarding complications of diabetes mellitus and more specifically neuropathy sample sizes were much smaller, ranging from 327 to 943 [30, 42, 43]. In [44] authors used a very large sample size (10,180) to train models using the MNSI variables as predictors, however again only results from internal cross-validation were reported. One study was carried out on a prospective cohort with single imputation using Random Forests and build a LR model with stepwise feature selection that achieved an AUC = 0.726 for the 3-year follow-up time-point. However, this estimate comes exclusively from internal validation [30]. In [42] authors trained a SVM to predict no, mild, moderate and severe DPN in a cross sectional cohort with an AUC = 0.76, however this is again an estimate based on internal validation only. Hyperglycaemia, hypertension, obesity, smoking, duration of diabetes and female gender have been identified as risk factors increasing the odds ratio for DPN [30, 31, 40, 45]. However, psychological factors have generally not been considered and importantly, only the presence of DPN was usually amongst the endpoints with no distinction between painful and painless neuropathy. ML has been used to predict co-morbid depression in people with diabetes mellitus, finding that female gender, having a higher number of diabetic complications and the presence of chronic pain were amongst the factors most highly correlated with major depression [32]. In this study we have used an array of demographics, clinical, quality of life and psychological features to classify painful or painless DPN in the largest (to the best of our knowledge) deeply phenotyped clinical cohort of people with painful or painless DPN (N = 1230) to date. ML was, applied in a realistic context that included the presence of missing values and different ways of defining the outcome, i.e. clinical diagnosis or questionnaire based.

## Methods

In this study we trained a diverse set of Machine Learning (ML) models to classify painful versus painless DPN in three datasets: a deeply phenotyped clinical cohort developed in the University of Oxford (5); Technion—Israel Institute of Technology; and Imperial College London. We then externally validated these models in a questionnaire-based phenotyped population cohort developed in the University of Dundee. We followed the TRIPOD/EQUATOR reporting guidelines [46], Additional file 1: Figure S1.

### Datasets

All data used in this study has been generated using the DOLORisk study protocol which has been described elsewhere [15]. The total sample size used for training and validating these models was 1230 people with diabetes mellitus, predominantly Type II.

Three large, deeply phenotyped, cross-sectional cohorts (DOLORisk Imperial College London, PINS—University of Oxford [5] and DOLORisk Technion – Israel Institute of Technology, N = 935) were used to train and estimate model’s performance using 5-times repeated tenfold cross-validation. Training datasets had deep clinical phenotyping. Participants were first screened for clinical neuropathy based on symptomatology and DPN was confirmed by abnormalities of nerve conduction studies or Intra Epidermal Nerve Fibre Density (IENFD) [47]. Neuropathic Pain (NeuP “pain caused by a lesion or disease of the somatosensory system”) was determined at the time of the clinical assessment according to the NeuP Special Interest Group (NeuPSIG) of the International Association for the Study of Pain (IASP) grading system [48].

The NeuPSIG grading for neuropathic pain was used to grade neuropathic pain.

This is pain with:

1. A distinct neuroanatomically plausible distribution, i.e. pain in symmetrically distributed in the extremities;
2. A history suggestive of a relevant lesion or disease affecting the peripheral or central somatosensory system—diagnosis of diabetes mellitus and a history of neuropathic symptoms including decreased sensation, positive sensory symptoms, e.g., burning, aching pain mainly in the toes, feet, or legs
3. Demonstration of the distinct neuroanatomically plausible distribution by at least one confirmatory test—presence of clinical signs of peripheral neuropathy, i.e., decreased distal sensation or decreased/absent ankle reflexes
4. Demonstration of the relevant lesion or disease by at least one confirmatory test—abnormality on either the nerve conduction tests or IENFD.

Possible neuropathic pain fulfils criteria 1 and 2. Probable neuropathic pain fulfils criteria 1, 2 and 3. Definite neuropathic pain fulfils all 4 criteria.

Participants with chronic (> 3 months) probable or definite NeuP were assigned to the NeuP group and participants with possible neuropathic pain were excluded. Participants with no pain or non-NeuP in the extremities were included in the no NeuP group [5, 15].

Models were externally validated in the independent GoDARTS – DOLORisk Dundee [49] dataset. This is a clinical cohort, phenotyped for pain and neuropathy using the DOLORisk protocol. GoDARTS participants with type 2 diabetes from Tayside, Scotland were re-phenotyped for neuropathic pain and related traits, by questionnaire, using the DOLORisk core protocol in order to be classified according to the presence and extent of neuropathic pain. A subset of the 1915 GoDARTS- DOLORisk Dundee participants could be classified as painful/painless DPN using validated questionnaires and screening questions for the presence and anatomical distribution of pain (N = 295), see Outcome Definition for more details. Data from these cohorts were collected between 2012 and mid-2019.

All variables that were common in both training and validation datasets and were missing in less than 50% of the training and validation datasets were considered as potential predictors. These include clinical, biochemical, demographical and self-reported quality of life data. A complete overview of the training and validation datasets is presented in Table 1. There was no significant difference in the outcome distribution between the training and validation sets. However, the age and Body Mass Index (BMI) of participants were lower in the training dataset and some self-reported quality of life and psychological variables were significantly different between the training and validation dataset. This is reflective of the fact that the training and validation cohorts are independent and comprised of different populations. PROMIS sleep disturbance t-score, Diabetes duration, Cholesterol, low-density lipoprotein (LDL), high-density lipoprotein (HDL), Creatinine and Triglycerides were removed due to high missing ratio (Additional file 1: Tables S1 and S2). The Chronic Kidney Disease (CKD) indicator variable was removed due to very low incidence, i.e. only 9 positives and 5 instances were removed due to very low HbA1c < 5 not consistent with diabetes mellitus.

**Table 1:** Descriptive summary statistics for all datasets

Dependent: Set Index		Train	Validation	Total	*p* value
Center	Dundee	0 (0.0)	295 (100.0)	295 (24.0)	< 0.001
	Imperial	180 (19.3)	0 (0.0)	180 (14.6)	
	Oxford	557 (59.6)	0 (0.0)	557 (45.3)	
	Technion	198 (21.2)	0 (0.0)	198 (16.1)	
EQ5D_Index	Median (IQR)	0.7 (0.6 to 0.8)	0.7 (0.5 to 0.8)	0.7 (0.5 to 0.8)	0.015
Depression_tscore	Median (IQR)	49.4 (42.2 to 56.8)	52.0 (41.0 to 58.7)	49.4 (41.0 to 57.5)	0.001
Anxiety_tscore	Median (IQR)	45.9 (37.1 to 56.4)	51.4 (40.3 to 57.5)	48.5 (40.3 to 56.4)	0.001
Sleep_Disturbance_tscore	Median (IQR)	56.3 (50.1 to 59.4)	51.8 (49.6 to 54.4)	53.3 (49.7 to 57.8)	< 0.001
Trauma	No	417 (44.6)	184 (62.4)	601 (48.9)	0.378
	Yes	194 (20.7)	99 (33.6)	293 (23.8)	
	(Missing)	324 (34.7)	12 (4.1)	336 (27.3)	
Hospital_stay	No	529 (56.6)	230 (78.0)	759 (61.7)	0.649
	Yes	81 (8.7)	31 (10.5)	112 (9.1)	
	(Missing)	325 (34.8)	34 (11.5)	359 (29.2)	
TIPIExtraversion	Median (IQR)	4.0 (3.0 to 5.0)	4.0 (3.0 to 5.0)	4.0 (3.0 to 5.0)	0.280
TIPIAgreeableness	Median (IQR)	5.0 (4.0 to 6.0)	5.0 (4.0 to 6.0)	5.0 (4.0 to 6.0)	0.531
TIPIConscientiousness	Median (IQR)	6.0 (4.5 to 6.5)	5.5 (4.5 to 6.5)	6.0 (4.5 to 6.5)	0.001
TIPIEmotionalStability	Median (IQR)	5.0 (4.0 to 6.5)	4.5 (4.0 to 6.0)	4.5 (4.0 to 6.5)	0.145
TIPIOpenness	Median (IQR)	5.0 (4.0 to 6.0)	4.5 (4.0 to 5.6)	4.5 (4.0 to 6.0)	0.069
Ever_smoked_status	No	312 (33.4)	116 (39.3)	428 (34.8)	0.001
	Yes	296 (31.7)	177 (60.0)	473 (38.5)	
	(Missing)	327 (35.0)	2 (0.7)	329 (26.7)	
Alcohol_consumption	Never	201 (21.5)	88 (29.8)	289 (23.5)	0.014
	Less than 1 day per month	119 (12.7)	75 (25.4)	194 (15.8)	
	1 to 3 days per month	74 (7.9)	50 (16.9)	124 (10.1)	
	1 or 2 days per week	102 (10.9)	34 (11.5)	136 (11.1)	
	3 or 4 days per week	44 (4.7)	24 (8.1)	68 (5.5)	
	Daily or almost daily	68 (7.3)	21 (7.1)	89 (7.2)	
	(Missing)	327 (35.0)	3 (1.0)	330 (26.8)	
Alcohol_consumption_likert	Median (IQR)	1.0 (0.0 to 3.0)	1.0 (0.0 to 3.0)	1.0 (0.0 to 3.0)	0.412
Alcohol_status	No	201 (21.5)	88 (29.8)	289 (23.5)	0.422
	Yes	407 (43.5)	204 (69.2)	611 (49.7)	
	(Missing)	327 (35.0)	3 (1.0)	330 (26.8)	
PCS_score	Median (IQR)	10.0 (3.0 to 23.0)	8.0 (4.0 to 20.0)	10.0 (3.0 to 22.0)	0.319
MNSI_score	Median (IQR)	5.0 (3.0 to 7.0)	4.0 (3.0 to 6.0)	5.0 (3.0 to 6.0)	0.146
DN4_score	Median (IQR)	4.0 (3.0 to 6.0)	3.0 (2.0 to 5.0)	4.0 (3.0 to 6.0)	< 0.001
Age	Median (IQR)	68.0 (60.0 to 74.0)	69.0 (63.0 to 77.0)	68.0 (61.0 to 74.0)	0.006
Gender	Female	300 (32.1)	104 (35.3)	404 (32.8)	0.335
	Male	634 (67.8)	190 (64.4)	824 (67.0)	
	(Missing)	1 (0.1)	1 (0.3)	2 (0.2)	
BMI	Median (IQR)	29.0 (26.0 to 32.9)	31.2 (27.8 to 35.5)	29.4 (26.2 to 33.5)	< 0.001
HBA1C	Median (IQR)	7.4 (6.6 to 8.5)	7.4 (6.7 to 8.7)	7.4 (6.7 to 8.5)	0.165
Diabetes_Duration	Median (IQR)	13.5 (8.0 to 20.0)	15.0 (12.0 to 20.0)	15.0 (11.0 to 20.0)	0.005
Cholesterol	Median (IQR)	4.1 (3.5 to 4.6)	3.9 (3.4 to 4.4)	3.9 (3.4 to 4.5)	0.141
LDL	Median (IQR)	2.1 (1.8 to 2.8)	2.0 (1.5 to 2.5)	2.0 (1.6 to 2.5)	0.159
HDL	Median (IQR)	1.2 (1.0 to 1.4)	1.1 (0.9 to 1.3)	1.1 (0.9 to 1.3)	0.018
Creatinine	Median (IQR)	75.0 (66.0 to 86.5)	80.0 (64.0 to 99.0)	79.5 (65.0 to 96.0)	0.124
TRIGLYCERIDES	Median (IQR)	1.7 (1.2 to 2.0)	1.8 (1.3 to 2.6)	1.7 (1.3 to 2.5)	0.041
CKD	No	926 (99.0)	272 (92.2)	1198 (97.4)	< 0.001
	Yes	9 (1.0)	23 (7.8)	32 (2.6)	
Outcome	Painful_neuropathy	617 (66.0)	181 (61.4)	798 (64.9)	0.166
	Painless_neuropathy	318 (34.0)	114 (38.6)	432 (35.1)	

Set index indicates the training or validation dataset. Numerical variables are represented by the median and Inter Quantile Range (IQR) in brackets, categorical variables by the absolute occurrence and percentage in brackets. Columns hold from left to right data for the Training, Validation and both datasets together. Right most column holds the p.value of the comparison between Training and Validation datasets using the chi-square test for categorical variables or the Kruskal Walis test for numerical variables. The number and rate of missing values is indicated for all factor levels

Independent variables include the 5-level EQ-5D-5L instrument [50] that comprises assessment of mobility, self-care, usual activities, pain/discomfort and anxiety/depression; the Patient-Reported Outcomes Measurement Information System (PROMIS) depression and anxiety measurement instruments [51]; a question assessing the experience of traumatic events before the age of 18 (Trauma); a question investigating whether someone had stayed in hospital for a long period because of a life threatening disease or situation before the age of 18; the extraversion, agreeableness, conscientiousness, emotional stability and openness personality dimension constructs from the ten-item personality inventory (TIPI) [52]; a self-reported ever smoked status; self-reported alcohol consumption in an ordered Likert scale; age; gender; BMI and sugar glucose levels (glycated haemoglobin HbA1c). Variables were filtered for zero or near zero variance numerical features, highly correlated features (> 0.8 Pearson's correlation coefficient) and factors with very low complexity, Additional file 1: Figure S2.

### Outcome definition

The outcome of this study was painful or painless Diabetic Peripheral Neuropathy (DPN). For the training datasets one or more physicians have defined phenotypes after detailed clinical examination and grading of neuropathic pain as discussed above and in line with IASP and NeuPSIG definitions [47, 53]. An overview of the training datasets including all independent variables is in Table 2.

**Table 2:** Descriptive summary statistics for the training dataset

Dependent: Outcome		Painful_neuropathy	Painless_neuropathy	Total	*p* value
Center	Dundee	1 (0.2)	3 (0.9)	4 (0.4)	< 0.001
	Imperial	134 (21.8)	43 (13.4)	177 (18.9)	
	Oxford	327 (53.3)	230 (71.7)	557 (59.6)	
	Technion	152 (24.8)	45 (14.0)	197 (21.1)	
EQ5D_Index	Median (IQR)	0.7 (0.5 to 0.7)	0.8 (0.7 to 0.9)	0.7 (0.6 to 0.8)	< 0.001
Depression_tscore	Median (IQR)	52.0 (44.7 to 59.4)	44.7 (38.2 to 50.9)	49.4 (42.2 to 55.9)	< 0.001
Anxiety_tscore	Median (IQR)	49.4 (40.7 to 58.4)	40.7 (37.1 to 50.8)	45.9 (37.1 to 56.4)	< 0.001
Sleep_Disturbance_tscore	Median (IQR)	56.3 (51.2 to 60.4)	54.3 (48.1 to 59.4)	56.3 (50.1 to 59.4)	0.016
Trauma	No	271 (44.1)	149 (46.4)	420 (44.9)	0.005
	Yes	146 (23.8)	45 (14.0)	191 (20.4)	
	(Missing)	197 (32.1)	127 (39.6)	324 (34.7)	
Hospital_stay	No	355 (57.8)	174 (54.2)	529 (56.6)	0.261
	Yes	60 (9.8)	21 (6.5)	81 (8.7)	
	(Missing)	199 (32.4)	126 (39.3)	325 (34.8)	
TIPIExtraversion	Median (IQR)	4.0 (2.5 to 5.0)	4.0 (3.0 to 5.0)	4.0 (3.0 to 5.0)	0.246
TIPIAgreeableness	Median (IQR)	5.0 (4.0 to 6.0)	5.0 (4.0 to 6.0)	5.0 (4.0 to 6.0)	0.345
TIPIConscientiousness	Median (IQR)	6.0 (4.5 to 6.5)	6.0 (5.0 to 7.0)	6.0 (4.5 to 6.5)	0.188
TIPIEmotionalStability	Median (IQR)	4.5 (3.5 to 6.5)	5.5 (4.0 to 6.5)	5.0 (4.0 to 6.5)	0.005
TIPIOpenness	Median (IQR)	5.0 (4.0 to 6.0)	4.5 (4.0 to 5.5)	5.0 (4.0 to 6.0)	0.654
Ever_smoked_status	No	203 (33.1)	108 (33.6)	311 (33.3)	0.150
	Yes	211 (34.4)	86 (26.8)	297 (31.8)	
	(Missing)	200 (32.6)	127 (39.6)	327 (35.0)	
Alcohol_consumption	Never	151 (24.6)	48 (15.0)	199 (21.3)	0.010
	Less than 1 day per month	82 (13.4)	37 (11.5)	119 (12.7)	
	1 to 3 days per month	52 (8.5)	23 (7.2)	75 (8.0)	
	1 or 2 days per week	68 (11.1)	34 (10.6)	102 (10.9)	
	3 or 4 days per week	25 (4.1)	19 (5.9)	44 (4.7)	
	Daily or almost daily	37 (6.0)	32 (10.0)	69 (7.4)	
	(Missing)	199 (32.4)	128 (39.9)	327 (35.0)	
Alcohol_consumption_likert	Median (IQR)	1.0 (0.0 to 3.0)	2.0 (1.0 to 4.0)	1.0 (0.0 to 3.0)	< 0.001
Alcohol_status	No	151 (24.6)	48 (15.0)	199 (21.3)	0.006
	Yes	264 (43.0)	145 (45.2)	409 (43.7)	
	(Missing)	199 (32.4)	128 (39.9)	327 (35.0)	
PCS_score	Median (IQR)	15.0 (6.0 to 27.0)	4.0 (0.0 to 11.0)	10.0 (3.0 to 23.0)	< 0.001
MNSI_score	Median (IQR)	6.0 (4.0 to 7.0)	3.0 (2.0 to 4.0)	5.0 (3.0 to 7.0)	< 0.001
DN4_score	Median (IQR)	5.0 (4.0 to 7.0)	2.0 (1.0 to 3.0)	4.0 (3.0 to 6.0)	< 0.001
Age	Median (IQR)	68.0 (59.0 to 73.0)	70.0 (63.0 to 75.0)	68.0 (60.0 to 74.0)	< 0.001
Gender	Female	206 (33.6)	92 (28.7)	298 (31.9)	0.156
	Male	408 (66.4)	228 (71.0)	636 (68.0)	
	(Missing)	0 (0.0)	1 (0.3)	1 (0.1)	
BMI	Median (IQR)	29.3 (26.0 to 33.3)	28.4 (25.5 to 32.2)	29.0 (26.0 to 33.0)	0.031
HBA1C	Median (IQR)	7.5 (6.7 to 8.7)	7.2 (6.5 to 8.2)	7.4 (6.6 to 8.5)	0.001
Diabetes_Duration	Median (IQR)	12.0 (6.0 to 17.0)	15.5 (9.2 to 23.8)	13.5 (8.0 to 20.0)	0.040
Cholesterol	Median (IQR)	4.0 (3.6 to 5.0)	4.2 (3.5 to 4.6)	4.1 (3.5 to 4.6)	0.460
LDL	Median (IQR)	2.3 (1.8 to 3.1)	1.9 (1.7 to 2.5)	2.0 (1.7 to 2.8)	0.186
HDL	Median (IQR)	1.1 (0.9 to 1.3)	1.2 (1.0 to 1.3)	1.2 (1.0 to 1.3)	0.692
Creatinine	Median (IQR)	75.0 (65.0 to 85.0)	80.0 (67.0 to 89.0)	78.0 (67.0 to 89.0)	0.735
TRIGLYCERIDES	Median (IQR)	1.7 (1.3 to 2.0)	1.5 (1.2 to 2.0)	1.7 (1.2 to 2.0)	0.514

Outcome variable indicates painful or painless DPN. Numerical variables are represented by the median and Inter Quantile Range (IQR) in brackets, categorical variables by the absolute occurrence and percentage in brackets. Columns hold from left to right data for people with Painful DPN, Painless DPN and the total of the Training datasets. Right most column holds the p.value of the comparison between Painful and Painless DPN using the chi-square test for categorical variables or the Kruskal Walis test for numerical variables. The number and rate of missing values is indicated for all factor levels

For the validation datasets we have used an array of structured, validated questionnaires and screening questions to define phenotypes. The Michigan Neuropathy Screening Instrument (MNSI) [54] questionnaire section alone, with a cut-off value of 3, was used to define diabetic neuropathy. Various cut-offs have been suggested for the MNSI clinical examination and questionnaire instrument [54–56]. In these it has been consistently reported that the cut-off of 7 for the questionnaire part when used in combination with clinical examination is too insensitive for stand-alone questionnaire use, whereas a cut-off score of 3 and above for the questionnaire only has been shown to have very good performance (AUC = 0.75, optimal cut-off > 2.0318) [54].

A screening question “Are you currently troubled by pain or discomfort, either all the time or on and off?” was used to define the presence of pain. Chronicity was screened using the question “How long have you been suffering with this pain or discomfort?” with a cut-off of > 3 months to define the temporal aspect of chronic pain. These questions have been validated and are identical to those used in previous population-based epidemiology studies of pain, including UK Biobank [57, 58]. Location of pain was assessed using the question “In the past three months; a) which of these pains was assessed using the question “In the past three months; a) which of these pains have you had, b) which one of these pains bothered you the most?” followed by a comprehensive choice of body locations including “Pain in your feet”. The participant was then asked to complete the self-complete version of the “Douleur Neuropathique en 4 Questions” (DN4) questionnaire [59] for the most bothersome pain were a score of 3 and above indicated the presence of NeuP. A definition of possible DPN required the presence of neuropathy as screened by the MNSI and chronic pain in the feet, regardless if it was the most bothersome pain present.

The DN4 questionnaire-only version was only considered to remove instances of conflicting evidence, i.e. painful neuropathy with a DN4 score under the cut-off value or painless neuropathy with a DN4 value over the cut-off, using a cut-off value of 3 to indicate the presence of NeuP. This cut-off has been validated to provide the optimal area under the Receiver Operating Characteristic curve (ROC) for the questionnaire-only section, i.e. excluding the clinical examination [59].

**Fig. 1:**
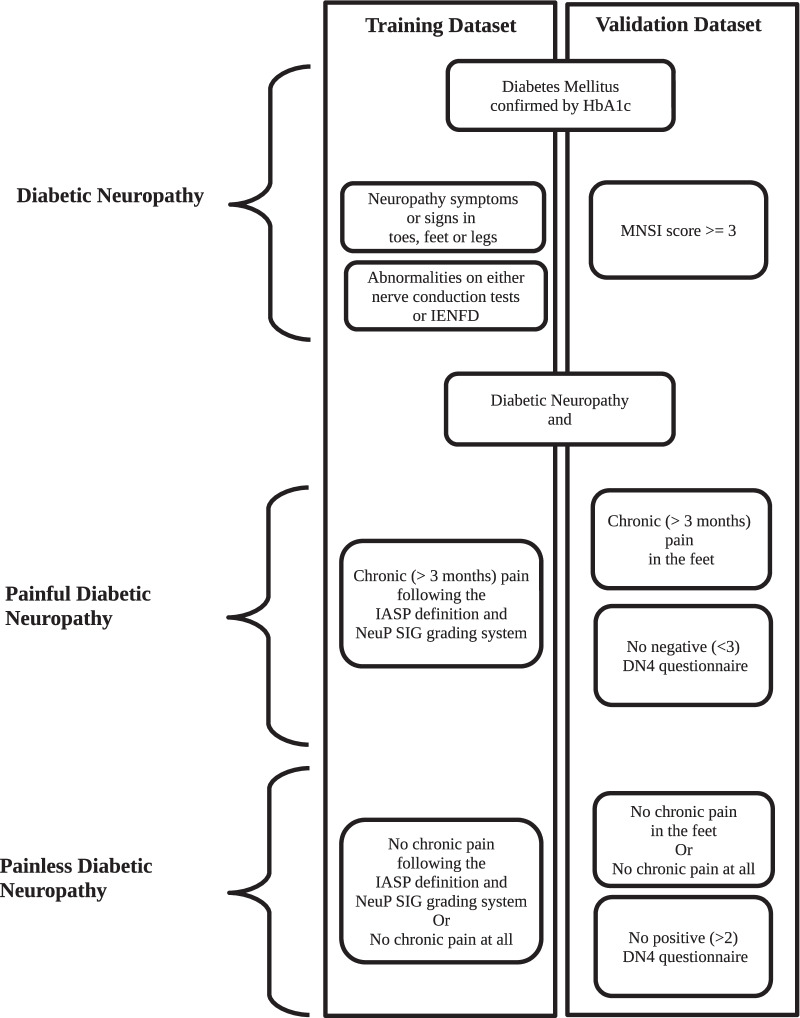
Flow diagram of the criteria used for outcome definition in the training and validation dataset

The presence of diabetes, neuropathy and chronic pain in the feet was used to define painful DPN. The presence of diabetes, neuropathy and no neuropathic pain in the feet defined the group of painless DPN, Fig. 1. An overview of the validation dataset is in Table 3.

**Table 3:** Descriptive summary statistics for the validation dataset

Dependent: Outcome		Painful_neuropathy	Painless_neuropathy	Total	*p* value
EQ5D_Index	Median (IQR)	0.6 (0.4 to 0.7)	0.7 (0.6 to 0.8)	0.7 (0.5 to 0.8)	< 0.001
Depression_tscore	Median (IQR)	54.7 (41.1 to 60.5)	48.9 (41.0 to 55.9)	52.1 (41.0 to 58.8)	< 0.001
Anxiety_tscore	Median (IQR)	51.7 (40.3 to 57.9)	48.3 (40.3 to 54.1)	51.4 (40.3 to 57.5)	0.001
Sleep_Disturbance_tscore	Median (IQR)	51.5 (49.4 to 54.2)	52.0 (49.7 to 54.5)	51.8 (49.6 to 54.4)	0.128
Trauma	No	107 (59.4)	73 (65.8)	180 (61.9)	0.289
	Yes	66 (36.7)	33 (29.7)	99 (34.0)	
	(Missing)	7 (3.9)	5 (4.5)	12 (4.1)	
Hospital_stay	No	144 (80.0)	82 (73.9)	226 (77.7)	0.269
	Yes	16 (8.9)	15 (13.5)	31 (10.7)	
	(Missing)	20 (11.1)	14 (12.6)	34 (11.7)	
TIPIExtraversion	Median (IQR)	4.0 (3.0 to 5.0)	4.0 (3.5 to 5.5)	4.0 (3.0 to 5.0)	0.077
TIPIAgreeableness	Median (IQR)	5.0 (4.0 to 6.0)	5.0 (4.5 to 6.0)	5.0 (4.0 to 6.0)	0.161
TIPIConscientiousness	Median (IQR)	5.5 (4.5 to 6.5)	5.5 (4.1 to 6.5)	5.5 (4.5 to 6.5)	0.600
TIPIEmotionalStability	Median (IQR)	4.5 (4.0 to 5.5)	5.0 (4.0 to 6.5)	4.5 (4.0 to 6.0)	0.027
TIPIOpenness	Median (IQR)	4.5 (3.5 to 5.5)	5.0 (4.0 to 6.0)	4.5 (4.0 to 5.5)	0.047
Ever_smoked_status	No	71 (39.4)	44 (39.6)	115 (39.5)	1.000
	Yes	108 (60.0)	66 (59.5)	174 (59.8)	
	(Missing)	1 (0.6)	1 (0.9)	2 (0.7)	
Alcohol_consumption	Never	60 (33.3)	27 (24.3)	87 (29.9)	0.358
	Less than 1 day per month	45 (25.0)	30 (27.0)	75 (25.8)	
	1 to 3 days per month	30 (16.7)	19 (17.1)	49 (16.8)	
	1 or 2 days per week	16 (8.9)	18 (16.2)	34 (11.7)	
	3 or 4 days per week	16 (8.9)	8 (7.2)	24 (8.2)	
	Daily or almost daily	11 (6.1)	8 (7.2)	19 (6.5)	
	(Missing)	2 (1.1)	1 (0.9)	3 (1.0)	
Alcohol_consumption_likert	Median (IQR)	1.0 (0.0 to 2.0)	1.0 (1.0 to 3.0)	1.0 (0.0 to 3.0)	0.134
Alcohol_status	No	60 (33.3)	27 (24.3)	87 (29.9)	0.130
	Yes	118 (65.6)	83 (74.8)	201 (69.1)	
	(Missing)	2 (1.1)	1 (0.9)	3 (1.0)	
PCS_score	Median (IQR)	11.0 (5.0 to 22.5)	5.0 (1.0 to 15.0)	8.5 (4.0 to 20.0)	< 0.001
MNSI_score	Median (IQR)	5.0 (4.0 to 6.0)	3.0 (3.0 to 4.0)	4.0 (3.0 to 6.0)	< 0.001
DN4_score	Median (IQR)	4.0 (3.0 to 5.0)	1.0 (0.0 to 1.0)	3.0 (2.0 to 5.0)	< 0.001
Age	Median (IQR)	68.0 (63.0 to 77.0)	69.0 (63.5 to 76.0)	69.0 (63.0 to 77.0)	0.590
Gender	Female	66 (36.7)	38 (34.2)	104 (35.7)	0.742
	Male	113 (62.8)	73 (65.8)	186 (63.9)	
	(Missing)	1 (0.6)	0 (0.0)	1 (0.3)	
BMI	Median (IQR)	31.4 (28.3 to 36.4)	30.8 (27.2 to 34.4)	31.2 (27.8 to 35.3)	0.204
HBA1C	Median (IQR)	7.5 (6.8 to 8.9)	7.4 (6.7 to 8.4)	7.4 (6.7 to 8.7)	0.311
Diabetes_Duration	Median (IQR)	15.0 (12.0 to 19.5)	16.0 (12.0 to 21.5)	15.0 (12.0 to 20.0)	0.921
Cholesterol	Median (IQR)	3.9 (3.5 to 4.4)	3.9 (3.4 to 4.4)	3.9 (3.4 to 4.4)	0.700
LDL	Median (IQR)	2.0 (1.5 to 2.5)	2.0 (1.6 to 2.5)	2.0 (1.5 to 2.5)	0.591
HDL	Median (IQR)	1.1 (0.9 to 1.3)	1.1 (0.9 to 1.3)	1.1 (0.9 to 1.3)	0.948
Creatinine	Median (IQR)	79.5 (64.0 to 99.0)	82.0 (64.5 to 98.0)	80.0 (64.0 to 99.0)	0 .568
Triglycerides	Median (IQR)	1.8 (1.4 to 2.7)	1.7 (1.3 to 2.4)	1.8 (1.3 to 2.6)	0.180

Outcome variable indicates painful or painless DPN. Numerical variables are represented by the median and Inter Quantile Range (IQR) in brackets, categorical variables by the absolute occurrence and percentage in brackets. Columns hold from left to right data for people with Painful DPN, Painless DPN and the total of the Validation dataset. Right most column holds the p.value of the comparison between Painful and Painless DPN using the chi-square test for categorical variables or the Kruskal Walis test for numerical variables. The number and rate of missing values is indicated for all factor levels

Datasets were not balanced as in both training and validation datasets the prevalence of painful is higher than the prevalence of painless diabetic neuropathy. 798 people had painful diabetic neuropathy (617 (66%) in training and 181 (61.4%) in validation datasets) and 432 had painless diabetic neuropathy (318 (34%) in training and 114 (38.6%) in validation datasets). Imbalance ratio for the training study is 0.52 and for the validation dataset 0.63. A sensitivity analysis assessing the change of the pooled coefficient estimates of a logistic regression model fitted on the imputed validation dataset showed no or very small sensitivity to various outcome definitions (Additional file 1: Figure S3). We further assessed the internal consistency of the validation cohort by calculating the Cohen’s Kappa inter-rater agreement between the MNSI question “Do you ever have any burning pain in your legs and/or feet?” and the response to the pain localisation question asking for “Pain in your feet”. We observed a significant (*p* value < 0.01), fair agreement between these responses, Kappa = 0.295.

### Missing values and feature construction

Datasets were largely harmonised and follow the DOLORisk core protocol [15]. However in the Oxford cohort, the Depression, Anxiety and Positive Outlook scale (DAPOS) [60] scores initially used have been replaced with the PROMIS [51] anxiety and depression short forms. Under the assumption that these constructs measured the same quantity in different scales, we linked DAPOS to PROMIS scores by scaling them together and then using the derived means and standard deviations to bring them in the same scale as PROMIS t-scores. Questions related to smoking were transformed to an “ever smoking” feature by taking into account the response to questions related to smoking at the time when the questionnaire was completed, clinical examination took place or in the past. The EQ-5D-5L [50] questionnaire was used alongside the UK normative data to obtain the EQ-5D index that was used as an independent variable. Alcohol consumption was transformed to a Likert type scale (0–5) using the following ordered levels: "Never", "Less than 1 day per month", "1 to 3 days per month", "1 or 2 days per week", "3 or 4 days per week", "Daily or almost daily". HbA1c was transformed to percentages (%) from mmol/mol when it was reported that way.

After removing all variables with > 50% of missingness, anxiety and depression t-scores experienced the highest rate of missingness (about 45%). The missing data mechanism was tested using the methodology in [61] for non parametric data against the null hypothesis that data is Missing Completely At Random (MCAR). Assessing whether missing data is dependent on observed data but not in missing values themselves, i.e. Missing At Random (MAR), is impossible as it requires knowledge of the missing values themselves. However we have visually assessed whether missing data was dependent on the outcome and other observed variables. Differences between the populations with missing/present data for all pairs of included variables were visualised using a matrix of plots and manually inspected for both the training and validation datasets. In tables providing dataset overviews we have included missing rates for each factor level. We then performed multiple imputations by chained equations using the predictive mean matching algorithm [62]. The number of imputations was set equal to the maximum missingness ratio experienced by any variable. Multiple imputation was done separately in the training and validation datasets to prevent information leakage between datasets. In order to accurately and not over-optimistically model the uncertainty introduced due to missing values we performed outcome agnostic imputation of the validation dataset. For this purpose a dataset was created by stacking both the training and imputation datasets and removing the outcome. The density plot of the observed and imputed values are in Additional file 1: Figures S4 and S5 for the training and validation datasets respectively.

### Statistical analysis

In an exploratory analysis before model fitting, dependencies between all independent variables and the outcome were assessed using the chi-square test for categorical data and the Kruskal Wallis test for numerical data comparison between two groups. Model’s performance was assessed by calculating overall accuracy as the proportion of the total number of correctly classified instances and the binomial test (*p* value < 0.05) was used to assess whether accuracy was higher than the prevalence of the majority class, i.e. no-information rate (NIR), the balanced accuracy as the mean of sensitivity and specificity and the Area Under the Precision/Recall Curve (AUPRC). However, all these metrics are sensitive to class imbalance and can lead to the selection of models that severely miss-classify the minority class. As we described above and is often the case in clinical cohorts both the training and validation datasets were not balanced (imbalance ratio 0.52 and 0.63 respectively). Therefore, we used the Mathews’ Correlation Coefficient (MCC) [63], which is similar to Pearson’s correlation coefficient, ranging from −1 to 1 and measures how correlated the prediction is to the true outcome. Moreover, MCC does not change with the substitution of the reference class as it is symmetrical and provides a more robust performance metric than accuracy, balanced accuracy and F1 score [63–65]. Model’s calibration was assessed on the validation set by visualizing the observed event percentage against the 10 prediction probability deciles. A linear model fitted to the calibration curves, i.e. event rate vs midpoint of the decile bin, was used to estimate the calibration slope and intercept.

Models were not updated nor calibrated after training in order to realistically assess the performance in predicting new data.

### Model training and validation

**Fig. 2:**
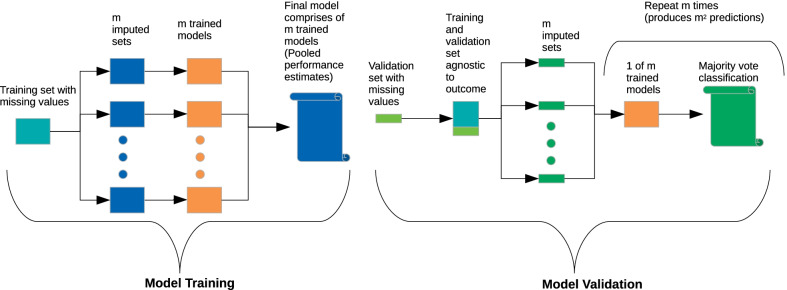
Block diagram of the training and validation workflow. During model training statistical learning algorithms use cross-validation to fine tune their internal parameters in order to maximise MCC and estimate their predictive performance in new un-observed data. Multiple imputation by chained equation was used to impute missing values in the training datasets. A model has been trained in each imputed dataset and aggregated in the final model ensemble. During model validation the ensemble of trained models is used to predict un-observed instances. After outcome agnostic multiple imputation all trained models are being used to predict the outcome in all imputed validation sets. Then results are aggregated by majority voting

We developed a workflow that uses the predict-then-aggregate strategy, after multiple imputation during both model training and validation. This way we modelled the uncertainty due to the imputation in both datasets, did not allow for information leakage between training and validation datasets and encapsulated all model building decisions in resampling and external validation [21]. Predictions were aggregated using majority voting, and point estimators were aggregated using the mean and Rubin’s rules to calculate the pooled—total standard deviation from the within/between imputations variance [22]. The workflow is visualised in Fig. 2. Models were trained on the training dataset using a maximum grid search of 60 tuning parameters optimised for the higher MCC. Numeric variables were centred and scaled for each cross-validation fold to ensure no information leakage between in and out-of-fold samples. The number of multiple imputations was equal to the highest rate of missingness observed across all variables. During training, we imputed missing values using all independent variables and the outcome. Thus we trained an ensemble of m = 45 models, optimised for the highest MCC during the 5-times repeated, tenfold cross-validation. The final model out-of-fold prediction probabilities, MCC, accuracy, balanced accuracy and AUPRC were calculated using Rubin’s rules. Partial dependence was also aggregated by calculating the mean marginal probability across imputations.

In validation, we first removed the outcome and pooled together the training and validation data. Then we performed m = 45 imputations as we did during training. The real outcome was kept unknown during the imputation of the validation set, simulating a real-world scenario of prediction of new data in the presence of missing values. Then we used the ensemble of trained models to predict the outcome on each of the 45 imputed validation datasets, producing 45^2^ predictions. Finally, these predictions were aggregated by majority voting.

We have trained a series of models using a set of diverged and well-known algorithms presenting the most significant subdomains of ML. We first benchmarked various algorithms, Additional file 1: figure S6, A, and selected the three best performers. Hierarchical clustering of the predicted class probabilities showed that these classifiers did not produce similar out-of-fold class probabilities and belonged in different clusters based on the dendrogram of class probabilities, Additional file 1: figure S6, B. The Random Forest [66] is an algorithm that produces an ensemble of decision trees using bagging, a technique that selects random subsets of potential predictors in order split each node of each growing tree. The Random Forest is also robust to the presence of multicollinearity of independent variables as a subset of predictors is randomly selected for each node split.

The Adaptive Regression Splines [67] is a multi-variable extension of regression that is able to model complex non-linear problems using an ensemble of simple linear functions that in aggregate optimise predictive ability. The algorithm has a built-in backwards elimination variable selection.

Finally, the Naive Bayes classifier as implemented in the e1071 package [68] is a probabilistic classifier that uses the Bayes’ rule to calculate the posterior probability of each class given a configuration of independent predictor variables.

In the case of Random Forests and Adaptive Regression Splines we trained an unweighted version and a weighted version with class weights inversely proportional to the class prevalences. Weighted models should match the probability distribution of the outcome closer than unweighted by having better calibration but could also run a higher risk of over-fitting training data and be less generalisable.

Models were trained in R [69] using the CARET package [70] and mltools [71]. Multiple imputation was done using MICE [62]. Marginal feature effects were calculated using the IML package [72], plots were rendered using ggplot2 [73] and tables using finalfit [74].

### Interpretability

In order to understand how independent variables values influenced models outcomes and to provide some interpretability of ML algorithms we calculated the variable importance in a model specific way and then scaled the metrics value to 100 to make them directly comparable. For the Random Forest we used the Gini importance index that calculates the mean decrease in impurity of the nodes produced by a split that uses a certain variable. For the Multivariate Adaptive Regression Splines we used the built-in backwards variable selection of the model to calculate the reduction in performance estimated by cross-validation when each variable is removed, and for the Naive Bayes model a ROC curve analysis was conducted for each variable.

Moreover, we have calculated, aggregated and visualised the marginal effects of all independent variables with a scaled importance of > 10 on each model’s outcome prediction. These Partial Dependence Plots (PDP) [26, 75] plots represent how the model’s outcome was influenced by changes in an independent variable values. Partial Dependence (PD) was calculated by marginalizing the classifier’s predicted probability over the distribution of the feature of interest.

PDPs not only show the marginal probability of the outcome given certain feature values but also provide an assessment on how robust and accurate is the information that a ML model can learn across the distribution of a feature’s values. A one-dimensional plot below each PDP shows the density of the feature values across each whole range. A dense distribution indicates that the PD can be accurately calculated for this range of values, while a sparse distribution shows that we cannot reliably calculate PD and also that there was probably not enough data in our training datasets for a model to learn a meaningful relationship for this feature range. We should also note that, while most ML models use different mechanisms to robustly handle some collinearity of features, PDP is sensitive to multicollinearity.

Partial dependence was aggregated for the whole ensemble of trained models using a customised function that calculated the mean across imputed datasets. In addition, trends of PDPs were estimated and visualised using a LOESS [76] smoothed curve. This analysis highlighted how the different values of each independent variable influenced the models predicted outcome all other things being equal.

## Results

### Performance estimates

Plot matrices of the differences between each pair of the included variables were visually assessed and showed no differences between participants with present/missing values in other variables and the outcome (Additional file 1: figures S7-S8). Moreover a non-parametric test of homoskedasticity and multivariate normality showed that the null hypothesis that data is MCAR could not be rejected at a *p* value < 0.01 in both the training (*p* value = 0.01) and test datasets (*p* value = 0.4). The combination of how the data has been generated, i.e. questionnaire based phenotyping and clinical examination, the marginally non-significant test for the training dataset and the visual assessment of missing values pairs plot indicate that missing data is likely to be MAR and thus can be robustly imputed using multiple imputation. During cross-validation the performance of the unweighted Random Forest had an MCC of 0.3489 (95% CI = 0.3446–0.3531) and the weighted Random Forest an MCC of 0.3396 (CI = 0.3354–0.3437). Regarding other scalar metrics, the AUPRC was 0.8116 (CI = 0.8098–0.8133) for the unweighted version and 0.8184 (CI = 0.8167–0.8201) for the weighted version, balanced accuracy was 0.6470 (CI = 0.6451–0.6490) for the unweighted and 0.6709 (CI = 0.6688–0.6730) for the weighted version.

Adaptive Regression Splines achieved similar performance. It had an MCC of 0.3327 (CI = 0.3286–0.3368) for the unweighed and 0.3248 (CI = 0.3207–0.3289) for the weighted model. AUPRC was 0.8207 (CI = 0.8191–0.8224) for the unweighed and 0.8188 (CI = 0.8171–0.8205) for the weighted model. Balanced accuracy was 0.6489 (CI = 0.6470–0.6509) and 0.6692 (CI = 0.6670–0.6713) for the unweighted and weighted model respectively.

Finally the Naive Bayes classifier had an MCC of 0.3112 (CI = 0.3070–0.3154), AUPRC of 0.8123 (CI = 0.8107–0.8140) and balanced accuracy of 0.6567 (CI = 0.6546–0.6588).

**Fig. 3:**
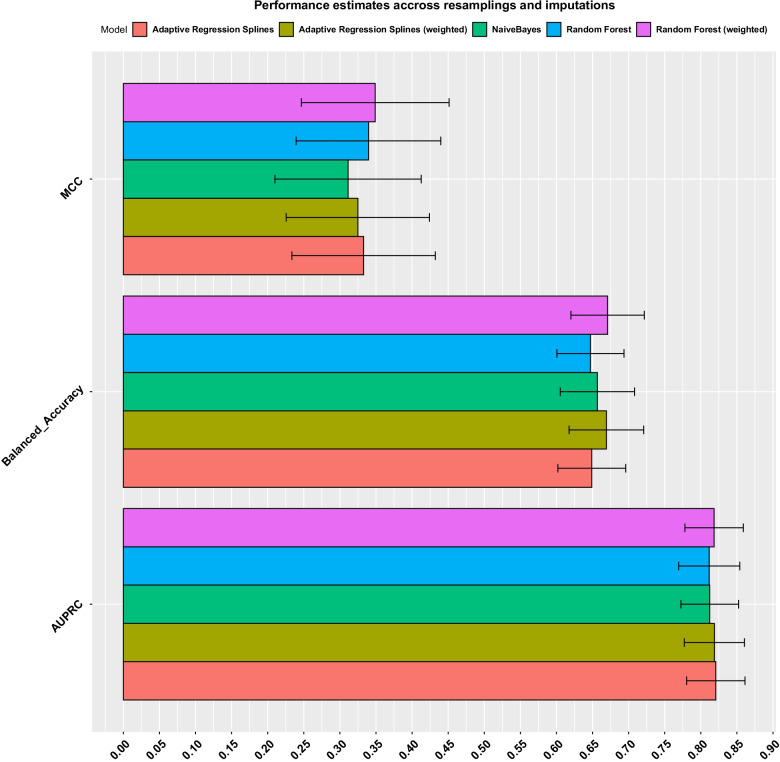
Performance estimates during model training, aggregated across resamplings and imputations. Solid coloured bars show the estimate mean and black lines the pooled SD calculated using Rubin’s rules. Visualised scalar metrics are the Mathews Correlation Coefficient (MCC), Balanced Accuracy and Area Under the Precision Recall Curve (AUPRC)

All models had good performance estimates indicating a moderate positive relationship between the outcome and models’ predictions, good balanced accuracy and very good AUPRC, Fig. 3. A ROC curve analysis is shown in Additional file 1: Figure S9.

### Variable profiling

**Fig. 4:**
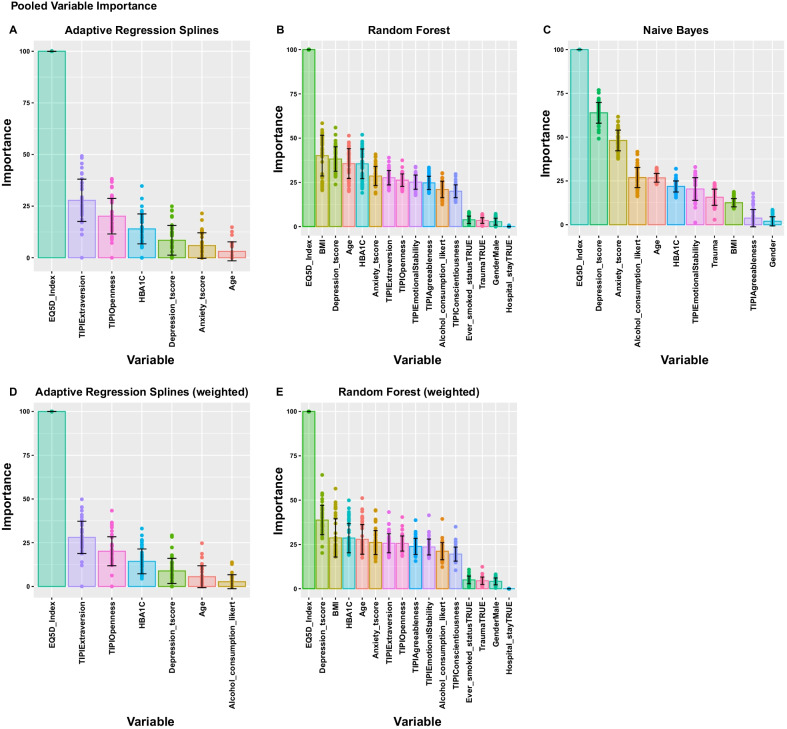
Pooled model specific variable importance across resamplings and imputations. Variable importance has been scaled from 0 to 100 to be directly comparable, coloured bars represent the mean, solid black lines the pooled SD calculated using Rubin’s rules, dots represent the variable importance estimated for each imputation. **A**,**C**: Mean reduction of classification accuracy when removing each independent variable in Adaptive Regression Splines. **B**, **E**: Gini coefficient that quantifies mean decrease of node impurity of the nodes produced by a split that uses each variable in a Random Forest. **C**: ROC analysis for each independent variable in Naive Bayes classifier

Model specific variable profiling revealed that a specific subset of variables were consistently amongst the most powerful predictors. These included quality of life, personality and psychology traits, age, and glucose control (Fig. 4). The built-in backwards feature elimination of the Adaptive Regression Splines algorithm revealed that the best performance was achieved when it considered the EQ5D index, TIPI extraversion and openness, HbA1c, Depression and Anxiety t-scores and age in descending order. EQ5D index, psychology and personality traits were always amongst the top predictors. Random Forest, based on the mean decrease on node impurity, ranked high the importance of BMI, Age and glucose control. Regarding modifiable lifestyle factors, Random Forest models also used alcohol consumption and smoking as predictive features, albeit with lower importance. Naive Bayes classifier produced a similar ranking with the addition of ranking alcohol consumption and experience of traumatic events before the age of 18 as having high importance. In all models, gender was not identified amongst the most powerful predictors although it was important enough to be included amongst the independent variables used in the final trained models. Weighted models had similar feature rankings to unweighted ones, with the noticeable difference of the inclusion of the alcohol consumption scale in the final model of the weighted Adaptive Regression Splines. We should also note that the Adaptive Regression Splines algorithm achieved its best performance using only 7 out of the 16 potential predictors.

### Feature effects

**Fig. 5:**
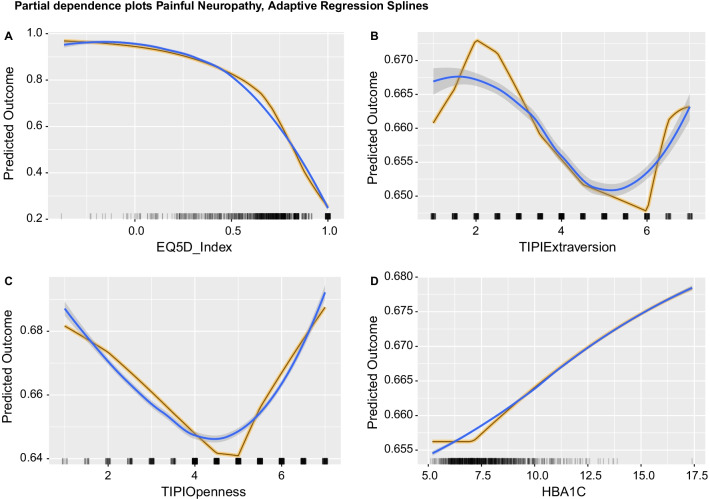
Partial Dependence (PD) plots for the Adaptive Regression Splines model aggregated across imputations. For panels **A**–**D**: Y-axis show the average marginal effect on the prediction given a certain value of the respective variable/feature. X-axis show the feature's values and the rug of marks under the x-axis indicate the density of data distribution across the feature’s full range. Black lines with yellow outline represent the aggregated PD. Blue lines show the fitted smoothed LOESS curve and highlight the PD trend. Selected independent variables had a scaled importance of > 10 and are arranged by decreasing importance value

The Adaptive Regression Splines classifier was more likely to predict painful DPN with lower EQ5D index (Fig. 5, A), indicating worse quality of life and showed a clear elbow around a cut-off threshold of 0.5, lower TIPI extraversion (Fig. 5, B) for most of the independent variable’s range, lower openness up to a value of 5 and then with increased prevalence for the top two openness values 6–7 (Fig. 5, C), and higher HbA1c (Fig. 5, D) indicating worse blood glucose level control.

**Fig. 6:**
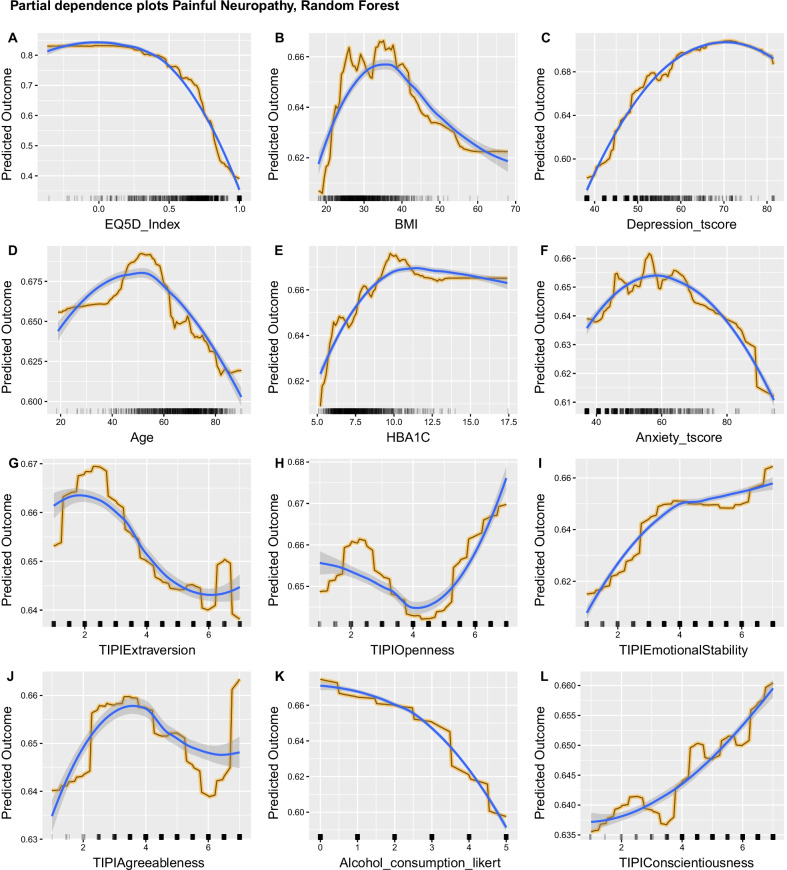
Partial Dependence (PD) plots for the Random Forest model aggregated across imputations. For panels **A**–**L**: Y-axis show the average marginal effect on the prediction given a certain value of the respective variable/feature. X-axis show the feature's values and the rug of marks under the x-axis indicate the density of data distribution across the feature’s full range. Black lines with yellow outline represent the aggregated PD. Blue lines show the fitted smoothed LOESS curve and highlight the PD trend. Selected independent variables had a scaled importance of > 10 and are arranged by decreasing importance value

The EQ5D index (Fig. 6, A) had the same influence on the Random Forests predictions. Higher BMI showed increased marginal probability for painful DPN (Fig. 6, B) for the part of the range where PD could be accurately calculated. The effect was non-monotonic for the part of the range where we had sparse density of values. Higher values of the PROMIS Depression t-score were associated with increased probability for a participant to be classified as having painful DPN (Fig. 6, C). Age (Fig. 6, D) had a similar non-monotonic effect as BMI. Ages 40–60 showed an increased marginal probability for painful DPN, while higher ages, where marginal probability was calculated with higher accuracy due to the higher density of values, showed a lower marginal probability for painful DPN. HbA1c (Fig. 6, E) showed again that worse blood glucose control increased the probability of a classification to the painful DPN group. Anxiety (Fig. 6, F) showed a similar influence to Depression for the part of the feature’s range that had high density of values. TIPI extraversion and openness (Fig. 6, G, H) were similar and had the same effect in Random Forest as in the Adaptive Regression Splines. Higher emotional stability increased the marginal probability of painful DPN (Fig. 6, I) and the same was observed for conscientiousness (Fig. 6, L). For the part of range where PD could be accurately estimated, higher TIPI agreeableness was negatively associated with painful DPN (Fig. 6, J). Higher alcohol consumption was consistently associated with decreased marginal probability of painful DPN (Fig. 6, K).

**Fig. 7:**
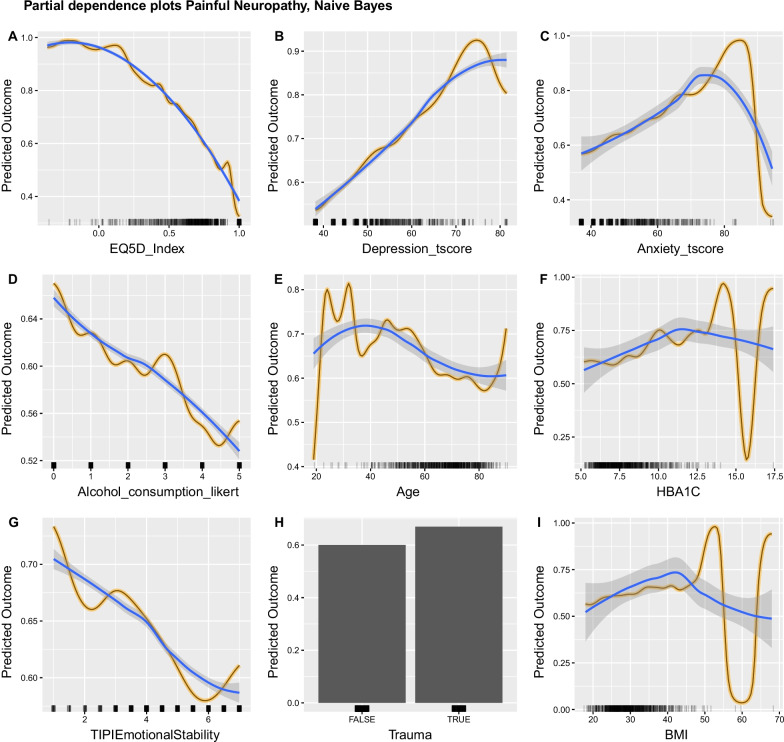
Partial Dependence (PD) plots for the Naive Bayes classifier aggregated across imputations. For panels **A**–**I**: Y-axis show the average marginal effect on the prediction given a certain value of the respective variable/feature. X-axis show the feature's values and the rug of marks under the x-axis indicate the density of data distribution across the feature’s full range. Black lines with yellow outline represent the aggregated PD. Blue lines show the fitted smoothed LOESS curve and highlight the PD trend. Selected independent variables had a scaled importance of > 10 and are arranged by decreasing importance value

The influence of EQ5D values in the Naive Bayes classifier (Fig. 7, A) was very similar with that which was observed in the Adaptive Regression Splines and the Random Forest. Depression and Anxiety had a strong effect on prediction (Fig. 7 B, C) and were both positively associated with increased marginal probability for painful DPN. Again alcohol consumption and Age were negatively associated with a prediction of painful DPN (Fig. 7, D, E). Higher HbA1c increased the marginal probability for painful DPN (Fig. 7, F). Finally, traumatic experiences under the age of 18 increased the marginal probability for painful DPN (Fig. 7, H). The same relationship between higher BMI values and higher probability for painful DPN was observed for the part of the BMI’s range where the model could learn meaningful relationships (Fig. 7, I). We should note that for most features, with the exception of EQ5D, Alcohol consumption and to an extent HbA1c, models showed unstable behaviour manifested with erratic changes in the marginal probabilities of the sparse values of the features’ range.

### Validation

**Fig. 8:**
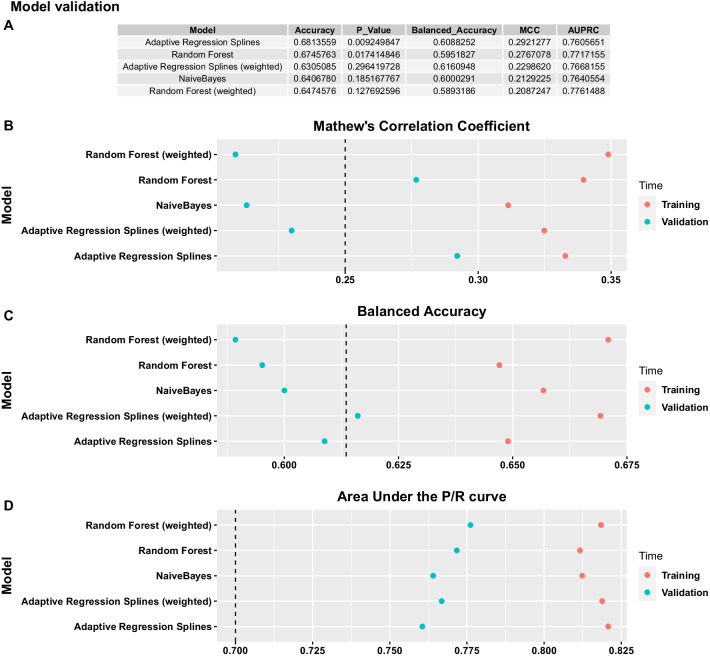
Model performance in the independent validation dataset. **A**: Scalar performance metrics. From left to right: Accuracy, the p.value of the negative binomial test with the null hypothesis that accuracy is not higher than the prevalence of the majority class, Balanced Accuracy, Mathews Correlation Coefficient, Area Under the Precision Recall Curve. **B**—**D**: Average performance estimates during training—red dots, versus performance achieved on the independent validation dataset—blue dots

Model validation took place in the independent cohort of 295 people. All ensembles of 45 strong trained classifiers predicted and aggregated prediction on all 45 imputed validation sets. Models’ performance was benchmarked using the MCC, AUPRC, balanced accuracy, accuracy and the p.value of the associated binomial test (Fig. 8, A). The AUPRC was very good for all models. However only the unweighted versions of Random Forest and Adaptive Regression Splines had an overall accuracy significantly better than the NIR (*p* value < 0.05) and were also the models that showed the highest MCC.

In general, performance was markedly reduced between training and validation (Fig. 8 B-D). Regarding the most important and robust metric MCC, Random Forest (0.28) and Adaptive Regression Splines (0.29) showed the smallest reduction in performance whilst still achieving good moderate positive correlation between predicted and observed outcomes. Additionally, considering MCC unweighted models markedly outperformed weighted ones (Fig. 8B–D).

**Fig. 9:**
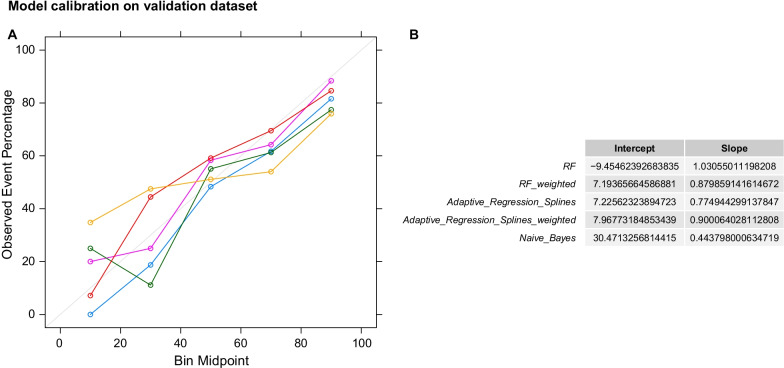
Model calibration in the independent validation dataset. **A**: Y-axis shows the observed event rate on the validation set. X-axis shows the predicted probability binned into 10 deciles. Colour coded lines show the event rate vs the midpoint of the probability decile for each model. **B**: The slope and intercept of a linear model fitted to the data of panel **A**, event rate = A*probability decile + b

Model calibration is presented in Fig. 9 A. Adaptive Regression Splines, Random forest, but not Naive Bayes, showed a monotonic increasing event rate for increased positive (painful DPN) class probabilities. The intercept and slope of the linear model fitted to calibration curves, i.e. event rate vs midpoint of decile bin, indicated good calibration for Random Forest and Adaptive Regression Splines, (Fig. 9, B). Intercept estimates showed that the former slightly over-fitted the data whereas the latter slightly under-fitted. However, the slope of both models was very good given that they had not been calibrated on the validation set. All models gained power moderately fast, requiring approximately 50% of the samples to correctly identify 60% of painful DPN events, (Additional file 1: Figure S10).

## Discussion

This study is one of the largest and most comprehensively phenotyped cohort of people with DPN, in which ML is applied to classify painful versus painless neuropathy. ML has been extensively used for the prediction and detection of diabetes mellitus in both cross sectional and prospective cohorts. Very high accuracies were only achievable by ensembles of diverse algorithms. Sample sizes varied a lot and most of times no external validation or calibration was carried out [34, 41]. Sample sizes are much smaller in the case of studies considering complications of diabetes mellitus. ML has been used in other studies focusing on diabetic neuropathic pain or neuropathy versus no pain or no neuropathy with smaller sample sizes ranging from 327 to 943 instances [42, 43]. In these studies no external validation was available, performance was mainly assessed by the AUC and imputation strategies were simpler. Our study achieved similar or better AUPRC > 0.75 for all classifiers on an independent validation datasets improving both in methodology and performance. Given the large effects of the presence of neuropathy we consider the distinction of painful vs painless DPN to be more challenging for a differential diagnosis, classification or prediction. Given this, we have managed to train models that had good to very good performance on an independent validation dataset.

Importantly the training and validation cohorts were collected at different sites, representing different populations and compiled using different methods. Nevertheless they all follow the core DOLORisk protocol, highlighting the importance of large harmonised cohorts.

This study used statistical learning techniques for painful/painless DPN in a realistic framework that accounts for the presence of missing values both during model training and when predicting new instances. There were no differences between pairs of included variables with missing versus present data, and the null hypothesis that data is MCAR could not be rejected, albeit marginally for the training dataset. These findings indicate that missingness is not related to the missing values themselves. However, given the nature of datasets, it is more likely that the mechanism of missingness is Missing At Random (MAR) rather than MCAR. In addition, we have taken measures to avoid over-estimating a model’s performance. The comparison of scalar metrics often used in statistical learning, including ML, highlights that the usage of accuracy or the AUC alone can significantly overestimate the model’s performance [77]. Importantly the two best models in the current study, i.e. Random Forests and Adaptive Regression Splines, showed the smallest reduction in performance in MCC between the training estimate and the validated performance, better calibration and an overall accuracy significantly better than the prevalence of the majority class. The fact that the models’ performances were reduced in validation highlights the importance of using an independent validation dataset. The optimistic performance estimates during model training are also known as over-fit, indicating a model with high variance that has learned nuances of the training dataset that are not necessarily generalisable to the new unknown instances. In addition, datasets that are imbalanced, as is often the case for clinical cohorts, where a condition of interest can be at the same time serious and rare, can also bias some performance metrics and produce highly optimistic estimates. This is a well-known issue in model benchmarking and validation [24, 64, 65] but is sometimes ignored in ML studies. Simpler and more parsimonious models are usually found to be more robust, trading off some variance with bias, and this could be the case with Adaptive Regression Splines, the algorithm that created the most parsimonious model in this study, and that also achieved the best performance in the validation dataset. Although a comprehensive comparison between different statistical learning techniques is beyond the scope of this paper, we should note that while it is technically doable to fit logistic regression models to any binary classification problem the assumptions that need to be made before fitting logistic regression and assessing the goodness of fit are rarely met in these types of datasets, i.e. linearity between independent variables and log odds and no multi-collinearity.

In the current study we have opted for using model-agnostic techniques to provide feature rankings and interpretability in order to highlight how independent variables’ values influenced the prediction probabilities of the trained models. Self-reported quality of life, psychological and personality traits have consistently been the most powerful predictors of painful DPN both in the current study and in previous studies using different modelling techniques [8, 30, 78]. BMI was a modifiable factor that was positively associated with painful DPN. Interestingly alcohol consumption is shown to be negatively associated with painful DPN. Although alcohol consumption has been found to be negatively associated with chronic pain [79], genetic randomisation had not found evidence supporting this protective effect and shown that most probably the direction of causality is that chronic pain reduces alcohol consumption [80]. Finally, age was found to have a complex non-monotonic relationship with the development of painful DPN. Although people with painful DPN are younger on average than those with painless DPN, this is mostly driven by a greater prevalence in the 40–60 age group. These kinds of complex relationships highlight the need of advanced statistical learning techniques in order to accurately model the development of painful or painless DPN. In addition, the sensitivity of PD on the density of the values of the respective feature highlights the need to use the largest possible clinical cohorts to allow ML models to learn meaningful relationships from data.

These models can be used either in the clinical context to assist patient stratification based on the risk of developing painful DPN if proven to be valid in a prospective study, or in the form of an online calculator that can return broad risk categories based on user input. Models’ performance and calibration prove that in both cases they can help timely diagnosis and prognosis, and could ultimately help patients and healthcare personnel to improve outcomes in those at highest risk by changing modifiable factors such as BMI and HbA1c control and institute earlier treatments including medication and/or psychological interventions. The fact that performance is moderate highlights the difficulty of classifying painful versus painless DPN without carrying out a face to face clinical assessment.

The main limitation of this study is the fact that the cohorts used were cross-sectional, therefore we cannot consider this a prognostic modelling study in the temporal sense. We also note that despite the fact that is the largest cohort that has been used to train models for the classification of painful/painless DPN the sample size is still smaller that what other ML studies have used towards different outcomes. However, the GoDARTS validation dataset is a longitudinal cohort and the PINS and DOLORisk Imperial cohorts are followed up for 2- and 5-year outcome re-phenotyping. We will use this data in the future to update and re-validate the models.

## Conclusions

ML models trained on large cross-sectional cohorts were able to accurately classify painful or painless DPN on an independent population-based dataset. Painful DPN was strongly associated with poorer self-reported quality of life, younger age, poor glucose control, high BMI and a number of psychological/personality factors. These models showed good performance in realistic conditions in the presence of missing values and noisy datasets.

## Supplementary Information

**Additional file 1**. Supplemental figures and tables.

## Data Availability

The datasets used and analysed during the current study are available from the corresponding author on reasonable request and under permission from the DOLORisk consortium. All scripts for data pre-process, model training, validation and Figure generation are available in http://github.com/gbaskozos/ML_DPN_Painful_Painless.

## Abbreviations

DPN: Diabetic Peripheral Neuropathy
ML: Machine Learning
PROMIS: Patient-Reported Outcomes Measurement Information System
TIPI: Ten-Item Personality Pnventory
BMI: Body Mass Index
HbA1c: Hemoglobin A1c
CKD: Chronic Kidney Disease
MNSI: Michigan Neuropathy Screening Instrument
DN4: Douleur Neuropathique en 4 Questions
DAPOS: Depression, Anxiety and Positive Outlook scale
NIR: No-information Rate
AUPRC: Area Under the Precision/Recall Curve
ROC: Receiver Operating Characteristic
MCC: Matthews’ Correlation Coefficient
PD: Partial Dependence
