# Supplementary material for: Classification of painful or painless diabetic peripheral neuropathy and identification of the most powerful predictors using machine learning models in large cross-sectional cohorts
Source: BMC Med Inform Decis Mak. 2022 May 29;22:144. doi: 10.1186/s12911-022-01890-x (PMC9150351; doi:10.1186/s12911-022-01890-x)
Supplement: Supplementary file 1 — Additional file 1. Supplemental figures and tables. [file 12911_2022_1890_MOESM1_ESM.pdf]

Supplemental Table 1

Variable	Missing_values
Center	0
EQ5D_Index	0.411764705882353
Depression_tscore	0.447058823529412
Anxiety_tscore	0.447058823529412
Sleep_Disturbance_tscore	0.565775401069519
Trauma	0.346524064171123
Hospital_stay	0.347593582887701
TIPIExtraversion	0.393582887700535
TIPIAgreeableness	0.394652406417112
TIPIConscientiousness	0.390374331550802
TIPIEmotionalStability	0.389304812834225
TIPIOpenness	0.39144385026738
Ever_smoked_status	0.349732620320856
Alcohol_consumption	0.349732620320856
Alcohol_consumption_likert	0.349732620320856
Alcohol_status	0.349732620320856
PCS_score	0.116577540106952
MNSI_score	0.381818181818182
MNSI_status	0.381818181818182
DN4_score	0.109090909090909
DN4_status	0.109090909090909
Age	0
Gender	0.00106951871657754
BMI	0.0117647058823529
HBA1C	0.0780748663101604
Neuropathy	0
Diabetes_Duration	0.86096256684492
Cholesterol	0.940106951871658
LDL	0.958288770053476
HDL	0.941176470588235
Creatinine	0.941176470588235
TRIGLYCERIDES	0.956149732620321
Depression_metric	0.447058823529412
Anxiety_metric	0.447058823529412
Outcome	0
Set_index	0

Proportions of missing values in training datasets

Supplemental Table 2

Variable	Missing_values
Center	0
EQ5D_Index	0.0687285223367698
Depression_tscore	0.0378006872852234
Anxiety_tscore	0.0515463917525773
Sleep_Disturbance_tscore	0.0240549828178694
Trauma	0.0412371134020619
Hospital_stay	0.116838487972509
TIPIExtraversion	0.0549828178694158
TIPIAgreeableness	0.0515463917525773
TIPIConscientiousness	0.0515463917525773
TIPIEmotionalStability	0.0652920962199313
TIPIOpenness	0.0515463917525773
Ever_smoked_status	0.00687285223367698
Alcohol_consumption	0.0103092783505155
Alcohol_consumption_likert	0.0103092783505155
Alcohol_status	0.0103092783505155
PCS_score	0.0790378006872852
MNSI_score	0
MNSI_status	0
DN4_score	0.549828178694158
DN4_status	0.549828178694158
Age	0.00343642611683849
Gender	0.00343642611683849
BMI	0
HBA1C	0.00687285223367698
Neuropathy	0
Diabetes_Duration	0.00343642611683849
Cholesterol	0
LDL	0.0446735395189003
HDL	0.00343642611683849
Creatinine	0.00687285223367698
TRIGLYCERIDES	0.013745704467354
Depression_metric	0
Anxiety_metric	0
Outcome	0
Set_index	0

Proportions of missing values in validation dataset

Supplemental Figure 1

Section/Topic	Item		Checklist Item	Page
**Title and abstract**				
Title	1	D;V	Identify the study as developing and/or validating a multivariable prediction model, the target population, and the outcome to be predicted.	1
Abstract	2	D;V	Provide a summary of objectives, study design, setting, participants, sample size, predictors, outcome, statistical analysis, results, and conclusions.	1
**Introduction**				
Background and objectives	3a	D;V	Explain the medical context (including whether diagnostic or prognostic) and rationale for developing or validating the multivariable prediction model, including references to existing models.	1-4
	3b	D;V	Specify the objectives, including whether the study describes the development or validation of the model or both.	5
**Methods**				
Source of data	4a	D;V	Describe the study design or source of data (e.g., randomized trial, cohort, or registry data), separately for the development and validation data sets, if applicable.	5-6
	4b	D;V	Specify the key study dates, including start of accrual; end of accrual; and, if applicable, end of follow-up.	6
Participants	5a	D;V	Specify key elements of the study setting (e.g., primary care, secondary care, general population) including number and location of centres.	5-6
	5b	D;V	Describe eligibility criteria for participants.	7
	5c	D;V	Give details of treatments received, if relevant.	NA
Outcome	6a	D;V	Clearly define the outcome that is predicted by the prediction model, including how and when assessed.	7
	6b	D;V	Report any actions to blind assessment of the outcome to be predicted.	8-9
Predictors	7a	D;V	Clearly define all predictors used in developing or validating the multivariable prediction model, including how and when they were measured.	8
	7b	D;V	Report any actions to blind assessment of predictors for the outcome and other predictors.	8-9
Sample size	8	D;V	Explain how the study size was arrived at.	8
Missing data	9	D;V	Describe how missing data were handled (e.g., complete-case analysis, single imputation, multiple imputation) with details of any imputation method.	8
Statistical analysis methods	10a	D	Describe how predictors were handled in the analyses.	8-9
	10b	D	Specify type of model, all model-building procedures (including any predictor selection), and method for internal validation.	9-10
	10c	V	For validation, describe how the predictions were calculated.	9-10
	10d	D;V	Specify all measures used to assess model performance and, if relevant, to compare multiple models.	9-11
	10e	V	Describe any model updating (e.g., recalibration) arising from the validation, if done.	9
Risk groups	11	D;V	Provide details on how risk groups were created, if done.	NA
Development vs. validation	12	V	For validation, identify any differences from the development data in setting, eligibility criteria, outcome, and predictors.	6
**Results**				
Participants	13a	D;V	Describe the flow of participants through the study, including the number of participants with and without the outcome and, if applicable, a summary of the follow-up time. A diagram may be helpful.	Figure 1
	13b	D;V	Describe the characteristics of the participants (basic demographics, clinical features, available predictors), including the number of participants with missing data for predictors and outcome.	Table 1
	13c	V	For validation, show a comparison with the development data of the distribution of important variables (demographics, predictors and outcome).	Table 1
Model development	14a	D	Specify the number of participants and outcome events in each analysis.	Table 1-3
	14b	D	If done, report the unadjusted association between each candidate predictor and outcome.	Table 1-3
Model specification	15a	D	Present the full prediction model to allow predictions for individuals (i.e., all regression coefficients, and model intercept or baseline survival at a given time point).	NA
	15b	D	Explain how to the use the prediction model.	15
Model performance	16	D;V	Report performance measures (with CIs) for the prediction model.	11, Figure 3, Figure 8
Model-updating	17	V	If done, report the results from any model updating (i.e., model specification, model performance).	NA
**Discussion**				
Limitations	18	D;V	Discuss any limitations of the study (such as nonrepresentative sample, few events per predictor, missing data).	14-15
Interpretation	19a	V	For validation, discuss the results with reference to performance in the development data, and any other validation data.	12-13
	19b	D;V	Give an overall interpretation of the results, considering objectives, limitations, results from similar studies, and other relevant evidence.	14-15
Implications	20	D;V	Discuss the potential clinical use of the model and implications for future research.	15
**Other information**				
Supplementary information	21	D;V	Provide information about the availability of supplementary resources, such as study protocol, Web calculator, and data sets.	16
Funding	22	D;V	Give the source of funding and the role of the funders for the present study.	16

*Items relevant only to the development of a prediction model are denoted by D, items relating solely to a validation of a prediction model are denoted by V, and items relating to both are denoted D;V. We recommend using the TRIPOD Checklist in conjunction with the TRIPOD Explanation and Elaboration document.

TRIPOD guidelines checklist

Supplemental Figure 2

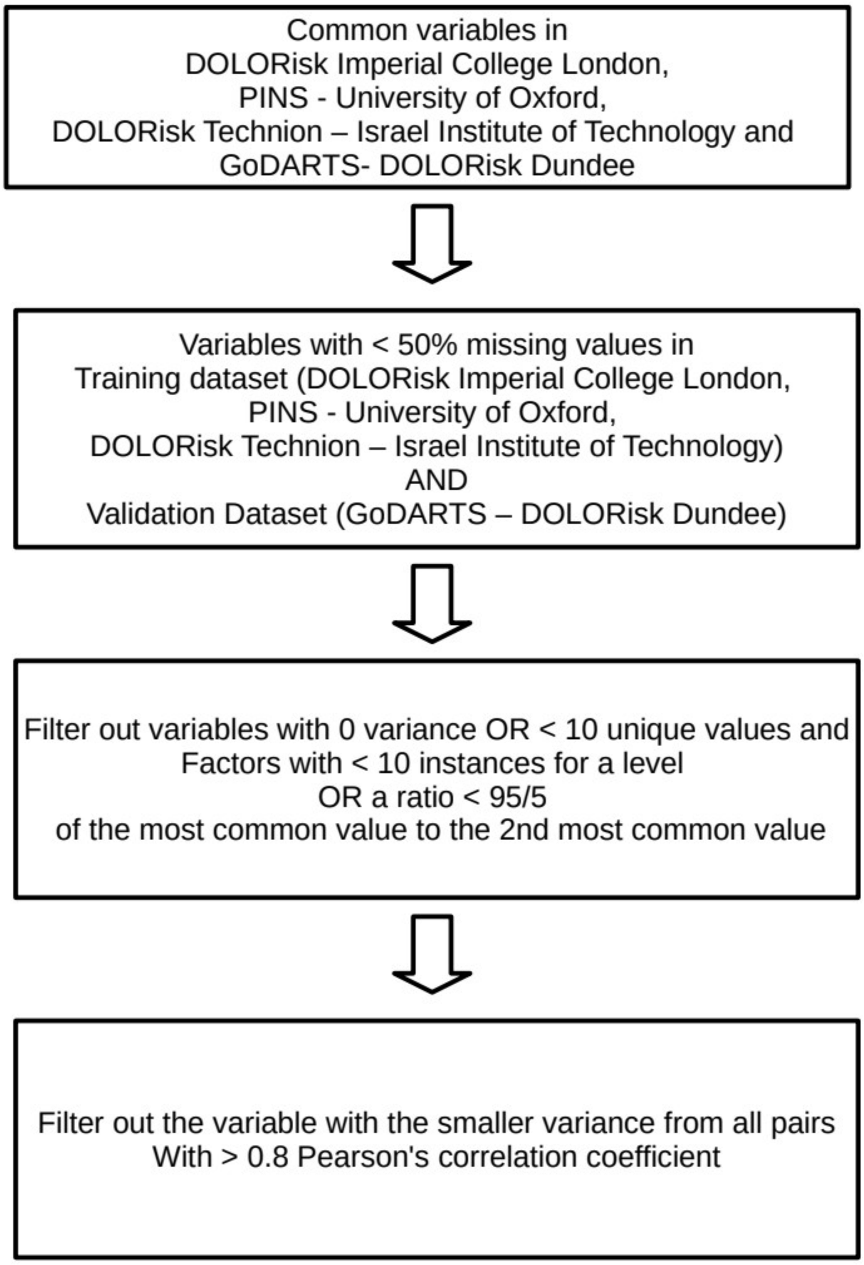

Flow chart showing the variable selection and data integration process.

Supplemental Figure 3

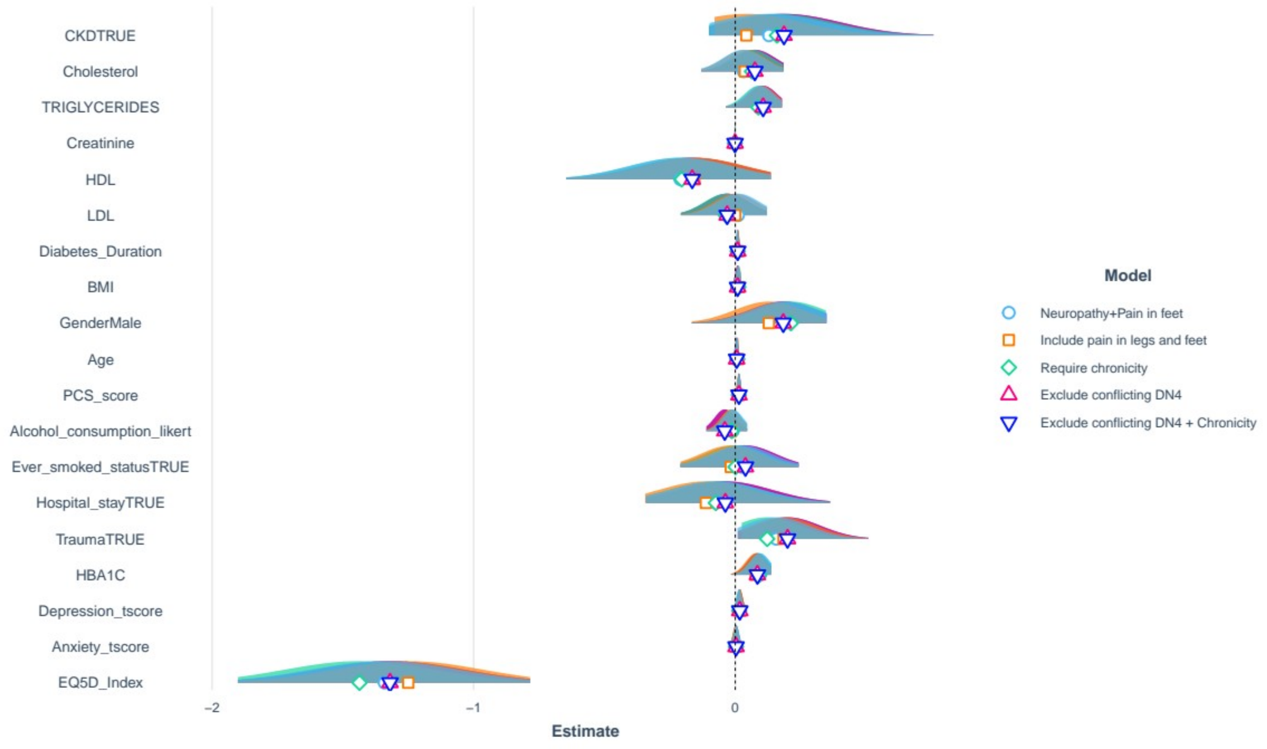

Sensitivity analysis regarding the outcome definition on the validation dataset. Missing values were imputed using multiple imputations by chained equation, then a logistic regression model was fitted and coefficients were pooled. We considered competent outcome definitions with the “Exclude conflicting DN4 + Chronicity” being the one used for the final outcome definitions. The shifts in the regression coefficients distributions shows that modelling is not highly sensitive to the different outcome definitions.

Supplemental Figure 4

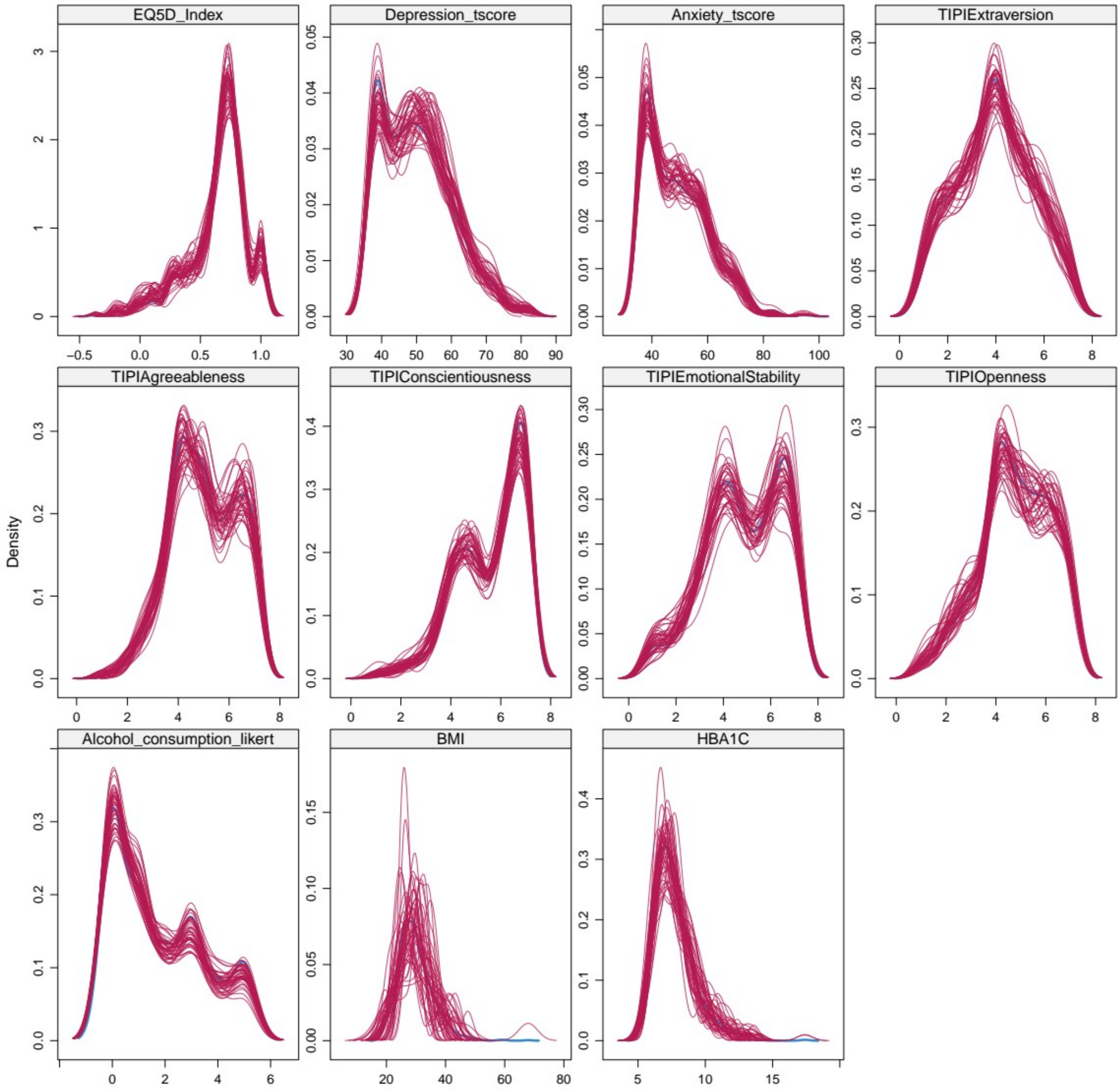

Density plots for the distribution of imputed values (pink lines) and present data points (blue lines) for the predictors with missing values on the training datasets.

Supplemental Figure 5

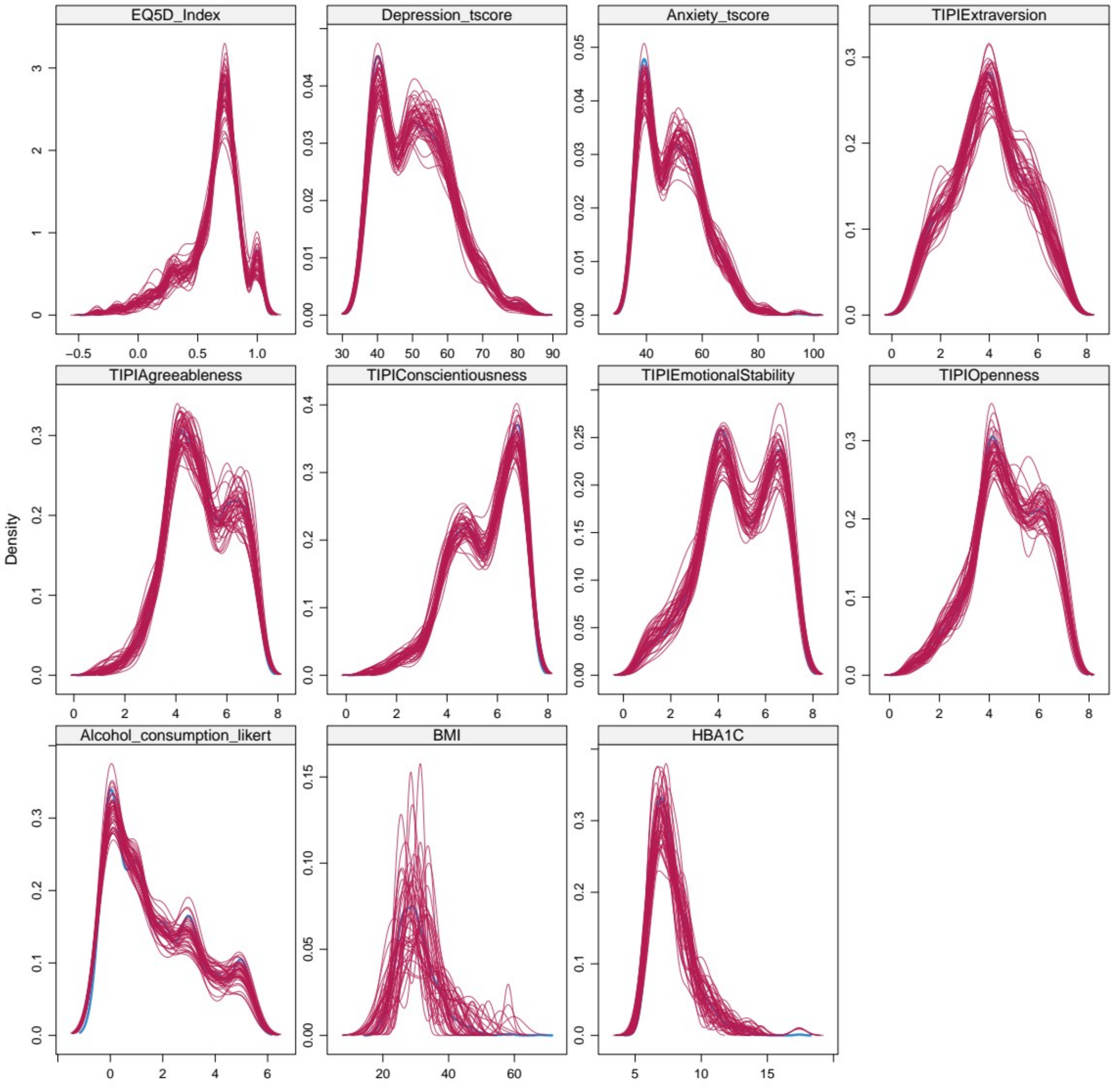

Density plots for the distribution of imputed values (pink lines) and present data points (blue lines) for the predictors with missing values on the validation dataset.

Supplemental Figure 6

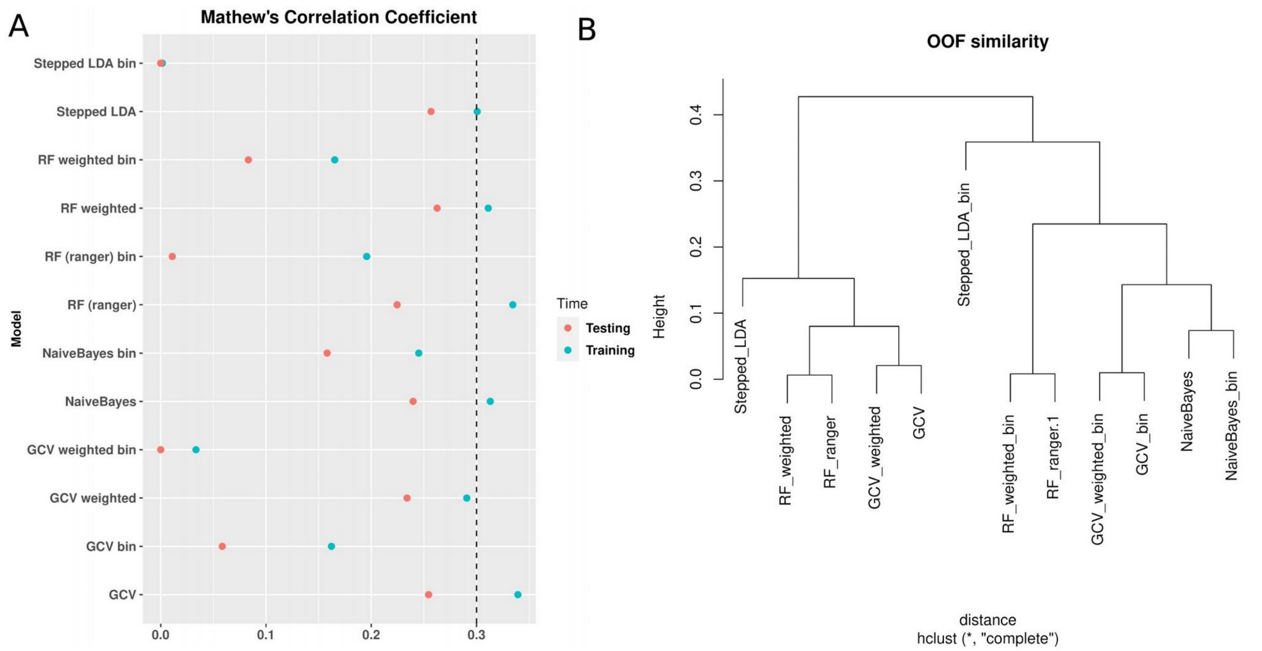

Benchmarking of various ML classifiers on the training and validation dataset. A) Average MCC during training – blue dots, versus performance achieved on the independent validation dataset – red dots. B) Hierarchical clustering of the out-of-fold class probabilities showing how dissimilar were the model's predictions during training.

Supplemental Figure 7

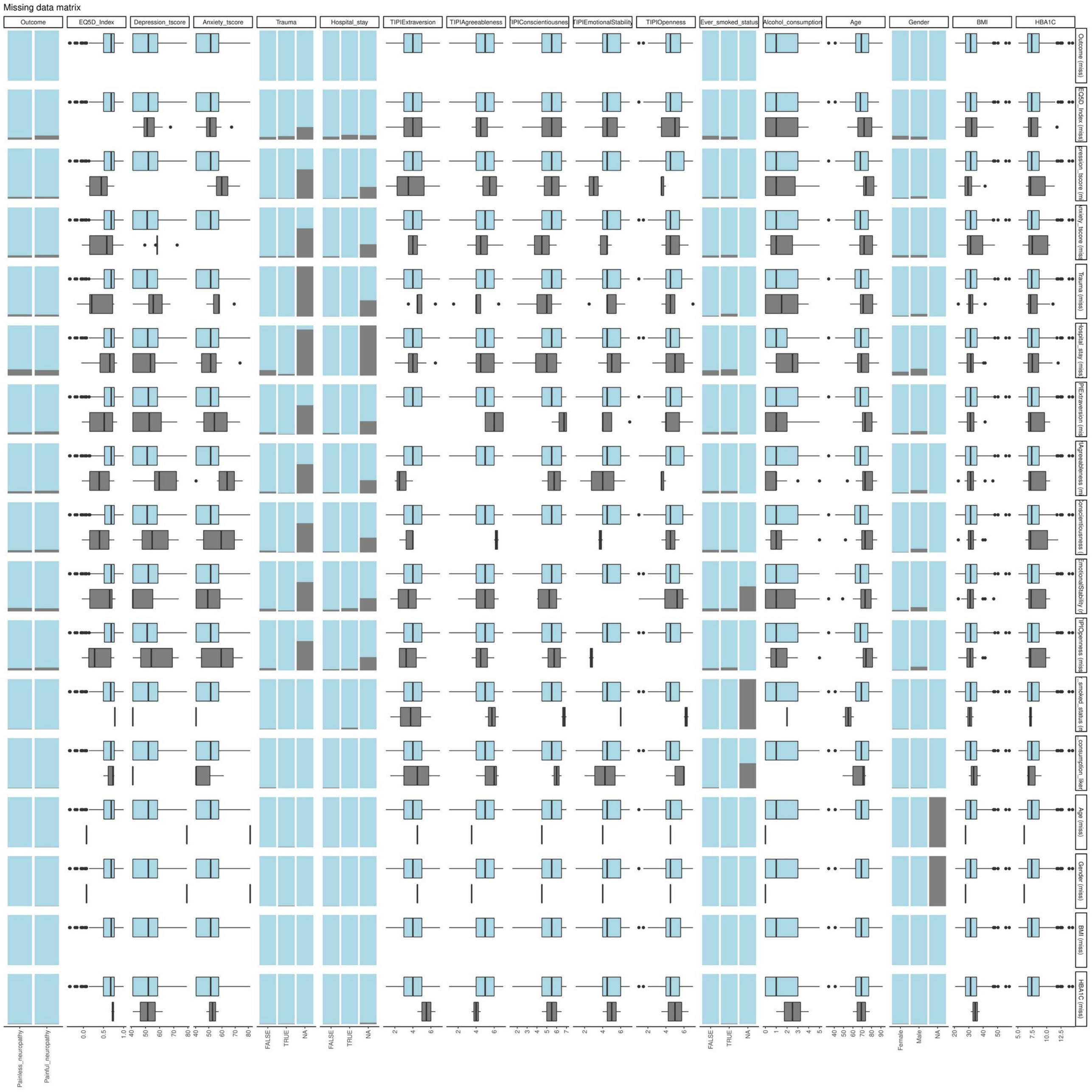

A plot matrix comparing missing values in all variables against each other for the training dataset. Missing values are indicated with grey, present with blue. In barplots the rate of missing values is presented in grey for each level of the respective factor. In boxplots the distribution of the respective variable for the population that has missing values on the other variable in presented as median and interquantile range of the green boxplot.

Supplemental Figure 8

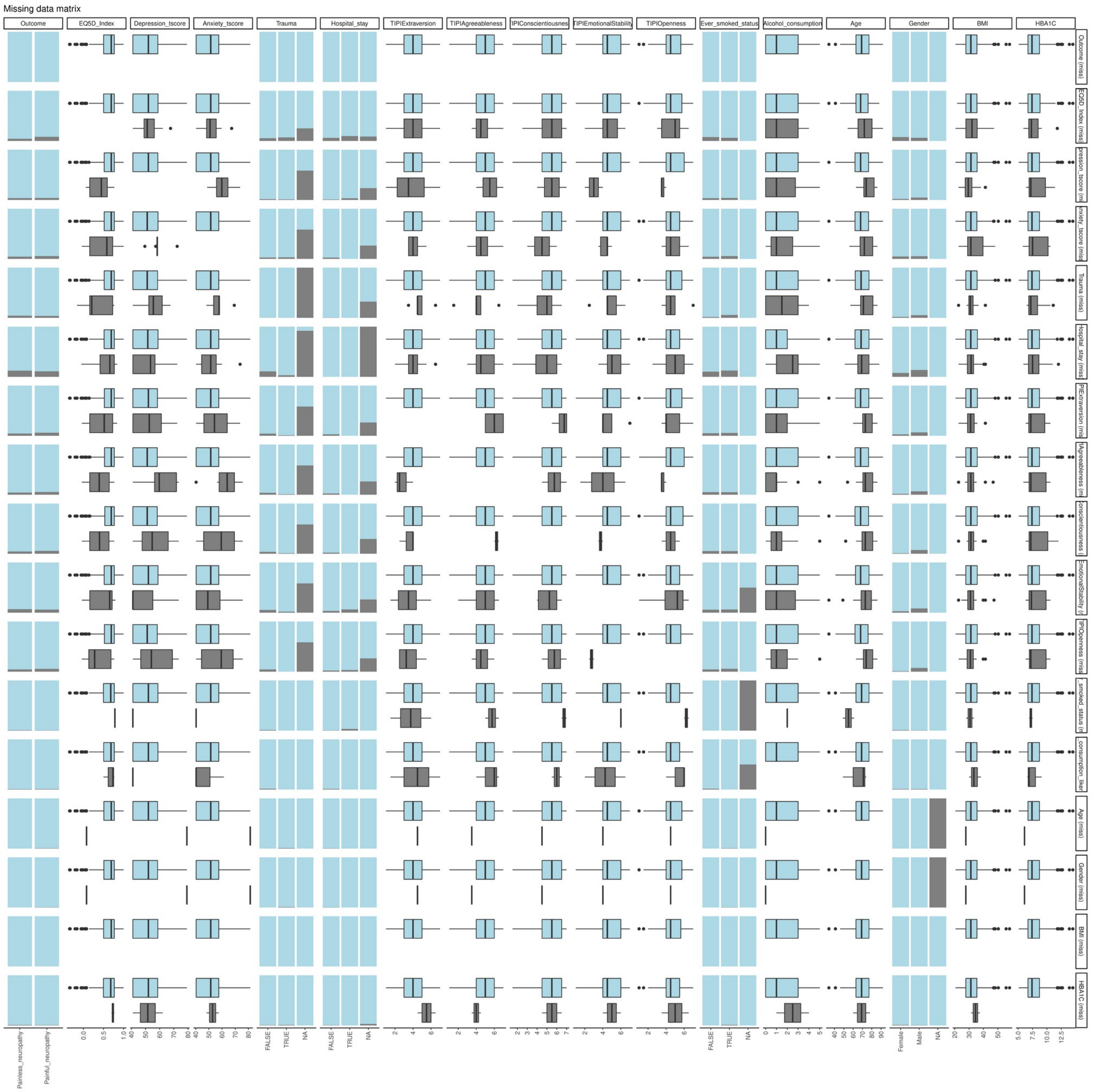

A plot matrix comparing missing values in all variables against each other for the validation dataset. Missing values are indicated with grey, present with blue. In barplots the rate of missing values is presented in grey for each level of the respective factor. In boxplots the distribution of the respective variable for the population that has missing values on the other variable in presented as median and interquantile range of the green boxplot.

Supplemental Figure 9

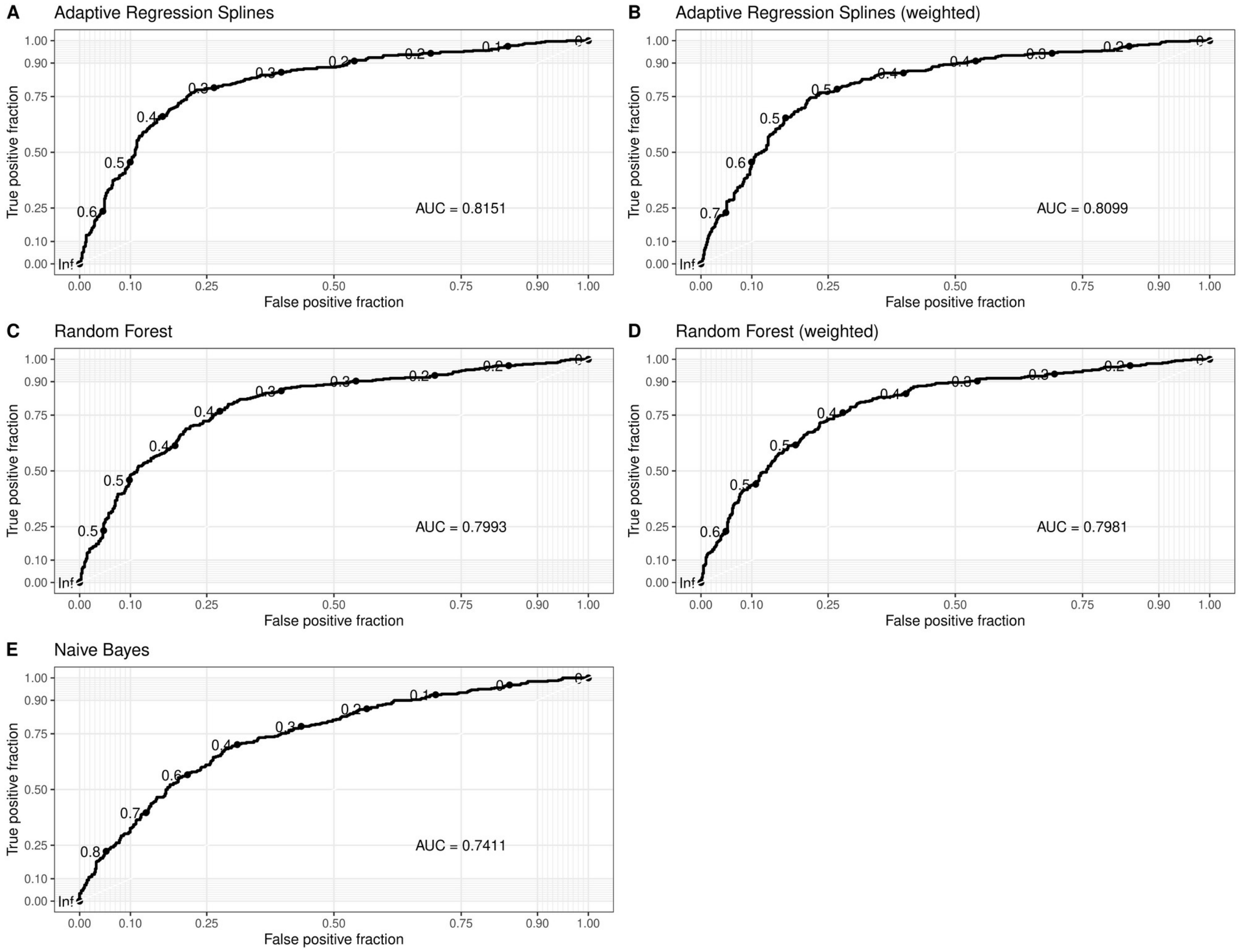

Receiver Operating Characteristic curves for the considered algorithms. Performance has been estimated with 5-times repeated 10-fold cross-validation. Probability cut-off values are shown in the curve alongside the calculated Area Under the Curve.

Supplemental Figure 10

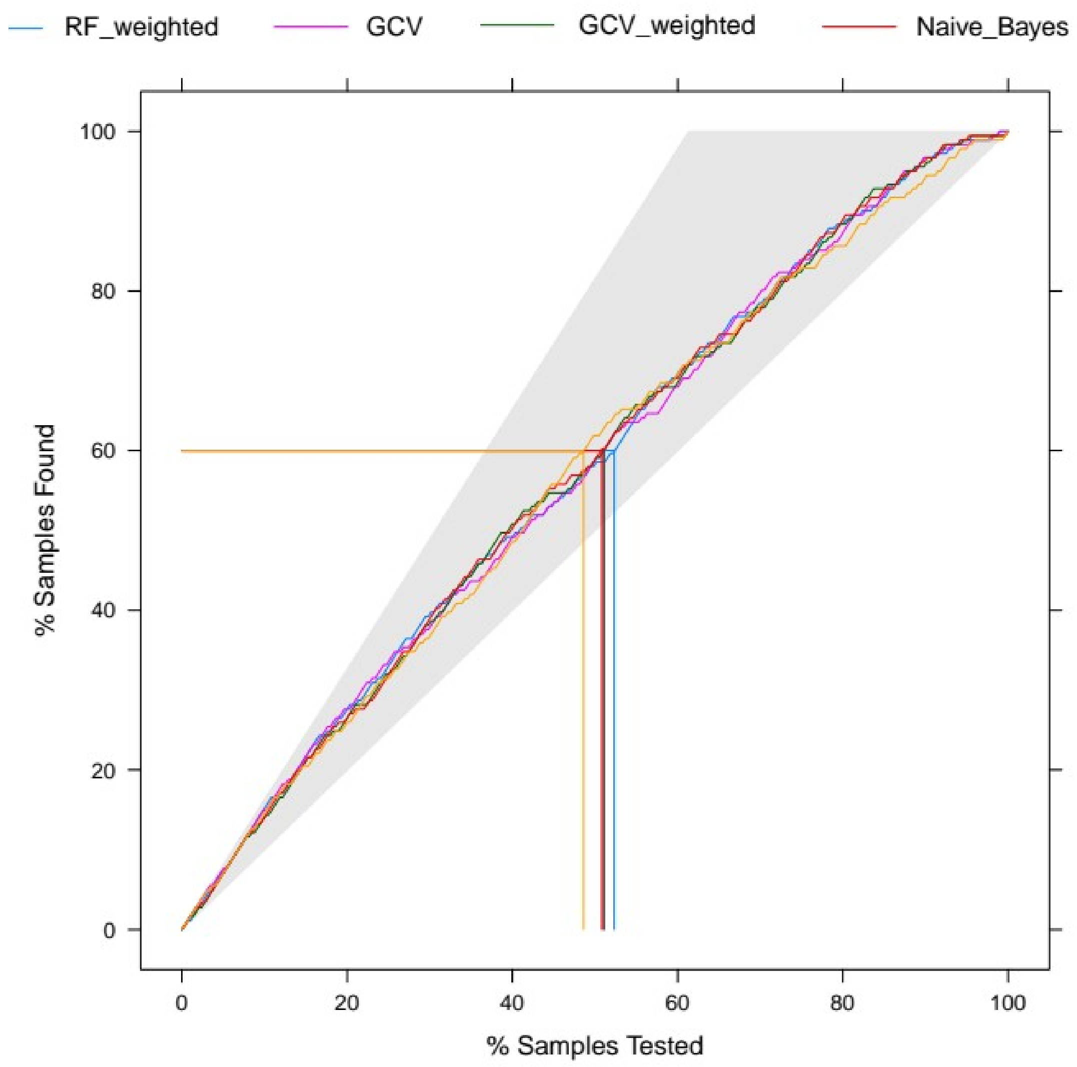

Lift curves showing how many samples (proportion) each model needs in order to correctly identify a certain proportion of true events (painful DPN).
